# SOPA and SIMPA: normalized single-sample integrated multiomics pathway analysis of tumor heterogeneity in solid cancers

**DOI:** 10.1093/bib/bbag338

**Published:** 2026-07-08

**Authors:** Hasan Alsharoh, Abdulrahman Ismaiel, George A Calin, Ovidiu L Pop, Ioana Berindan-Neagoe, Andreas Bender

**Affiliations:** Doctoral School of Biomedical Sciences, Faculty of Medicine and Pharmacy, University of Oradea, Str. Universitatii Nr.1, 410087 Oradea, Romania; Department of Genomics, MEDFUTURE Institute for Biomedical Research, Iuliu Haţieganu University of Medicine and Pharmacy, Str. Gh. Marinescu Nr.23, 400337 Cluj-Napoca, Romania; 2nd Department of Internal Medicine, Iuliu Haţieganu University of Medicine and Pharmacy, Str. Clinicilor 3-5, 400006 Cluj-Napoca, Romania; Department of Cancer Biology, The University of Texas MD Anderson Cancer Center, 1515 Holcombe Boulevard, Unit 422, Houston, TX 77030, United States; Translational Molecular Pathology, MD Anderson Cancer Center, Texas State University, 1515 Holcombe Blvd, Houston, TX 77030, United States; The RNA Interference and Non-coding RNA Center, MD Anderson Cancer Center, Texas State University, 1515 Holcombe Blvd, Houston, TX 77030, United States; Faculty of Medicine and Pharmacy, University of Oradea, P-ta 1 December 10, 410073 Oradea, Romania; Department of Genomics, MEDFUTURE Institute for Biomedical Research, Iuliu Haţieganu University of Medicine and Pharmacy, Str. Gh. Marinescu Nr.23, 400337 Cluj-Napoca, Romania; Doctoral School, Iuliu Hatieganu University of Medicine and Pharmacy, Victor Babes Nr.8, 400012 Cluj-Napoca, Romania; Academy of Medical Sciences, Bulevardul I.C. Braitanu Nr.1, Sector 3, 030171 Bucharest, Romania; Department of Genomics, MEDFUTURE Institute for Biomedical Research, Iuliu Haţieganu University of Medicine and Pharmacy, Str. Gh. Marinescu Nr.23, 400337 Cluj-Napoca, Romania; Department of Medicine and Center for Biotechnology, College of Medicine and Health Sciences, Khalifa University of Science and Technology, Abu Dhabi, Shakhbout Bin Sultan Street, Hadbat Al Za'faranah, Zone 1, 12778, United Arab Emirates; STAR-UBB Institute, Babeş-Bolyai University, Str. Mihail Kogălniceanu Nr.1, 400084 Cluj-Napoca, Romania; Centre for Molecular Informatics, Department of Chemistry, University of Cambridge, Lensfield Road, Cambridge CB2 1EW, United Kingdom

**Keywords:** personalized pathway analysis, systems biology, integrated multiomics, single sample analysis, gene set enrichment analysis, immune-metabolic dysregulation

## Abstract

Inter-sample tumor heterogeneity poses significant challenges to metastatic cancer treatment. Although multiomics analyses provide nuanced molecular insights into tumor heterogeneity, current molecular pathway analysis tools focus on group-based comparisons, which may overlook differential single-sample perturbations. Here, we present normalized single-sample single-omic pathway analysis (SOPA), and its extension, normalized single-sample integrated multiomics pathway analysis (SIMPA), as a bioinformatics pipeline for performing supervised differential pathway analysis. The pipeline utilizes custom algorithms to analyze differential pathway activity in single samples, comparing the molecular profile in each sample to a range of controls. In single -omics analysis, SOPA shows advantages compared to standard tools such as single sample gene set enrichment analysis and gene set variation analysis in identifying single sample deviations from predefined controls. For integrated multiomics, we show that in predefined-control contexts, SIMPA provides an effective alternative over unsupervised tools such as multiomics gene set analysis (MOGSA) and PAthway Deviation scores using Multiple Factor Analysis (padma), addressing tumor heterogeneity. Particularly, SIMPA unveiled particular tumor subgroups with dysregulated immune and metabolic pathway activity marked by variable immune infiltration and survival differences, which were missed by MOGSA and padma. Overall, SOPA and SIMPA are valuable in the supervised analysis of single samples and allow for the investigation of complex multiomics data to gain personalized hypothesis-generating insights. The flexibility of this pipeline allows implementation in preclinical and clinical research, offering significant advantages over prior pathway analysis tools in studying systems biology. The Python package for SOPA and SIMPA is freely accessible at https://github.com/hasanalsharoh/SIMPApy/.

## Introduction

Metastatic tumors have high inter-sample heterogeneity, which led recent literature to move from standard analysis of RNAseq and other single -omic data to more complex integrated multiomics (IM) [1]. One of the most commonly used tools for analyzing biological pathway activity in single -omics research is gene set enrichment analysis (GSEA) [2]. GSEA generates pathway (or gene set) insights by summarizing data from differential gene activity. However, one limitation GSEA suffers from is its focus on group-based differences, which may result in analyses that show trends between groups of samples, but overlook single sample perturbations. Understanding single sample perturbations allows us to gain more insights into cancer subgroup-specific progression and to design personalized therapies. To address this, other tools emerged, such as single sample GSEA (ssGSEA) [3], and gene set variation analysis (GSVA) [4], which enable the analysis of single -omics, particularly RNAseq, in single samples. More recent tools, such as multiomics gene set analysis (MOGSA) [5] and PAthway Deviation scores using Multiple Factor Analysis (padma) [6], allow to conduct IM pathway analysis on single samples.

While single sample analysis tools were designed to unravel disease heterogeneity [3–5], there are several major limitations that remain to be addressed in the methodology of ssGSEA, GSVA, and MOGSA. Firstly, ssGSEA uses within-sample absolute expression to rank genes, which may hinder between-sample comparability [3]. On the other hand, GSVA utilizes a density function to create a “control sample,” which is then used as reference to compare single samples to [4]. Thus, GSVA does not provide the possibility to directly conduct supervised comparisons between predefined cohorts. Moreover, the single -omic tools do not output $P$-values in their standard implementations, and offer no false-positive control. Importantly, the majority of tools for pathway analysis are designed for RNA sequencing (RNAseq) analysis. As for MOGSA, similarly to GSVA, it does not allow to conduct supervised comparisons, which may hinder the preservation of group-based trends when analyzing single samples [5]. Padma uses unsupervised multiple factor analysis (MFA) to calculate pathway deviation scores, yet it can be utilized for projecting samples onto a reference space to show deviation from predefined populations [6]. Nonetheless, to the best of our knowledge, padma was not validated for supervised analyses.

**Figure 1:**
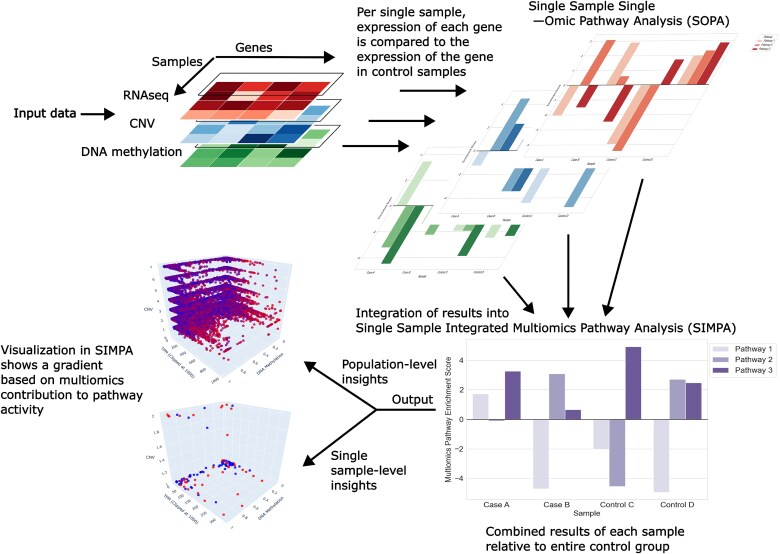
Workflow of the SOPA and SIMPA pipeline showing input data from RNAseq, CNV, and DNAm are processed by SOPA to compare each gene in a single sample to a control population, then the results are integrated by SIMPA to produce population-level and single-sample-level multiomics pathway enrichment scores.

Here, we developed a bioinformatics pipeline, termed single sample single-omic pathway analysis (SOPA), and its IM extension, single sample IM pathway analysis (SIMPA). SOPA utilizes custom ranking metrics developed for RNAseq, copy number variation (CNV), and DNA methylation (DNAm) data analysis. Below in Fig. 1 we summarize the methodology of SOPA and SIMPA. Our custom ranking metrics for single -omics implement control-normalization, which compares each gene in a single sample to the corresponding gene across the control population. Therefore, our custom metrics deliver the capability to conduct supervised analyses for single samples in predefined control groups. Additionally, since IM analyses provide more detailed analyses than single -omic studies [1, 7, 8], SIMPA utilizes integrated RNAseq, CNV, and DNAm pathway analysis results to deliver system-level insights into differential pathway analysis.

In this work, we aimed to show the capability of SOPA and SIMPA, implementing a case-control design of late-stage cancers with metastatic activity (TMAs), and early-stage tumors without metastatic activity (TWAs) on populations curated from the Cancer Genome Atlas (TCGA). Additionally, we highlight the potential of supervised analyses of single samples through SOPA compared to unsupervised standard tools (ssGSEA and GSVA) in TCGA datasets and external datasets obtained from the gene expression omnibus (GEO). Further, we investigate the performance of SIMPA against MOGSA and padma in the TCGA IM dataset. Finally, we utilize SIMPA to uncover immune-metabolic dynamics relevant in metastatic progression, missed by MOGSA, by investigating early and advanced tumor subgroups identified through the SIMPA pipeline. Therefore, we establish SIMPA as a valuable tool to conduct single sample pathway analysis for datasets with predefined controls utilizing IM data.

## Materials and methods

### Data resources, collection, filtering, and imputation

We used a custom search filter to query TCGA, including solid cancers with over 1000 samples, and collected RNAseq, CNV, and DNAm data. Further, we filtered and excluded 3D organoids, metastatic tumors (non-primary tumor samples), and blood samples. We used the GDC-Client Python script using the manifest files collected from the search query to download the data. Additionally, we downloaded patients’ clinical information when available. To manage the large datasets, we used the Dask v2024.9.1 python package. The clinical data included mapped sample case IDs, age, days to last follow up, days to death, alive status, cancer location, American Joint Committee on Cancer staging, and Tumor, Node, Metastasis (TNM) classification. All of the study datasets, their description, and the number of samples assessed are shown in Table 1. Methylation sites in the included datasets were mapped to gene names using the Illumina Methylation EPIC manifest v1.0b5.

**Table 1:** Datasets used in this study, with details of the number of samples found for each dataset from the respective source.

**Dataset**	**Total samples**	**Description**	**Source**
**The cancer genome atlas**			
RNAseq	6666	Platform: Illumina Workflow: STAR—counts Data: Raw counts and TPM	https://portal.gdc.cancer.gov/ Curated DOI: (raw data and manifest) https://doi.org/10.5281/zenodo.20067447
CNV	4100	Platform: Affymetrix SNP6.0 Workflow: Absolute Liftover Data: CN, gene start and end locations	https://portal.gdc.cancer.gov/ Curated DOI: (raw data and manifest) https://doi.org/10.5281/zenodo.20067447
DNAm	4817	Platform: Illumina Methylation EPIC, Illumina Methylation 450k Workflow: SeSAMe Methylation Beta Estimation Data: Beta values	https://portal.gdc.cancer.gov/ Curated DOI: (raw data and manifest) https://doi.org/10.5281/zenodo.20067447
**Gene expression omnibus**			
RNAseq	77	Platform: Illumina Data: Normalized counts	GEO accession: GSE165252
CNV	34	Platform: Affymetrix OncoScan™ FFPE Express V2 334K Data: GISTIC2.0 calls	GEO accession: GSE39280
DNAm	290	Platform: Illumina Methylation 450k Data: Beta values	GEO accession: GSE207460

Exact datasets can be found in the linked DOIs.

The multiomics datasets obtained from TCGA were subsequently processed. RNAseq data was filtered for genes with 0 total counts across all samples. As for CNV data, as per prior studies, we removed genes with $>50\%$ missing values across the total population, and used K-nearest neighbors (KNN) through the knnimpute v0.1.0 python package to impute the rest of the values [9]. KNN was found to perform best on similar data types at a parameter of five neighbors. For DNAm data, methylation sites with $>20\%$ missing values were removed, and the rest were imputed through the SoftImpute module in the FancyImpute v0.7.0 python package. The performance of KNN was found not to be as robust as other tools such as MissForest, and SoftImpute in DNAm beta values imputation [10]. However, as MissForest requires significantly higher computational resources for modest performance improvement, we chose SoftImpute for beta value imputations, due to the size of the DNAm dataset. Previous research indicated that using these imputation tools does not introduce significant bias in the data [9, 10]. To further ensure our results can be generalized, we used the external datasets from the GEO database without applying any filtering or imputation.

### Study group design and clinical data integration

Genomic alterations have been characterized throughout cancer pathological progression stages [11–13]. TNM staging classifies the pathological progression of tumors based on tumor size (T), nodal involvement (N), and metastasis (M) [14]. Thus, we utilized TNM staging to define metastatic progression in this study as tumor transition from N0M0 (designated TWAs) to N1+ or M1+ (designated TMAs), as implemented in a previous preprint [15]. Although different papers compared “early” and “late” stage cancer, the distinction threshold for early and late stages was not as thoroughly defined as in our implementation [16–18]. Here, we opted for this design of early-stage cancer (TWAs)—late stage cancer (TMAs), as it could potentially allow for accurate assessment of the molecular aspects governing metastatic transition in solid cancers. The specific criteria used to determine TWA/TMA designations for each sample is provided in Supplementary Method 1.

To validate the suitability of this TWA-TMA study design to provide insights into metastatic progression, we first assessed survival differences between TWAs and TMAs using the log-rank test and Kaplan-Meier curves (significance threshold at $P<.05$) through the lifelines python package v0.29.0 [19]. Survival analysis was only conducted for samples with tumor pathological stage data available, and samples without tumor staging data were excluded from subsequent analyses. Secondly, we performed exploratory data analysis across all omics to identify differentially expressed genes, frequently mutated genes, and differentially methylated sites and genes. The exploratory data analysis was performed as preliminary validation to establish whether significant molecular differences exist between TWAs and TMAs.

To identify significantly differentially expressed genes, we used pyDESeq2 v0.4.10, a DESeq2 implementation in python on RNAseq raw counts [20]. Significance threshold was considered at Benjamini–Hochberg (BH) corrected $P<.05$, and an absolute Log2 fold change $>0.5$ [21]. For frequently mutated genes, we used Fisher’s test, through the scipy v1.14.0 [22] python package, to compare frequencies between cases and controls, and copy numbers (CN) of genes were categorized to gain ($CN>2$), neutral ($CN=2$), and loss ($CN<2$) (this categorization is used in the literature [23]). Significant frequencies were determined at BH corrected $P<.05$. For differential methylation analysis, $M$-value regression absolute beta difference $>0.2$ (used in the literature [24]), and BH-corrected $P<.05$ in methylation were considered the significance thresholds. Numpy v1.23.5 was used to perform the calculations [25].

### Multiomics data integration strategy

To integrate the multiomics data, several considerations were to be made, as we opted to perform a gene-centric integration of the RNAseq, CNV, and DNAm-omics. Gene-centric integration allows for direct inter-omics comparisons between pathways with predefined genes. For RNAseq and CNV data, TCGA provides gene names mapped to transcripts and genomic regions, respectively.

A particular challenge in gene-centric integration of DNAm data is that methylation sites vary in their annotations [26], and thus require further consideration. The literature shows that different methylation sites may be annotated to the same gene, while some found in promoter regions may be mapped to multiple genes. Particularly, gene-body methylation has been shown to be largely independent from promoter methylation across different species [27, 28]. Similarly, to summarize the level of methylation for a gene, combining both promoter and gene-body methylation would cause confounding effects, and may not be representative of the gene’s methylation status. Due to the conflicting annotations of promoter methylation sites, and that gene-body-related methylation sites carry considerable information on genes [29], we decided to filter DNAm data to gene-body methylation sites.

Statistical approaches to summarize gene-level methylation vary in the literature. In the original gene-centric padma implementation [6], the authors used a single probe that carried the most variability to represent gene-level methylation. Nonetheless, this approach leads to a large loss of information from discarding all other methylation sites, and may not be biologically representative. While other tools have attempted to average methylation beta values from sites mapped to similar genes [30], recent recommendations strongly suggest using weighting strategies, although the optimal weighting strategy remains under debate [31, 32]. As per recommendations [33], we conducted linear regression analysis through the statsmodels v0.14.2 python package [34] on $M$-values. Further, we used resulting $P$-values as weights to calculate the weighted average beta-values for methylation sites mapped to the same genes. This approach allows for the prioritization of significantly methylated sites when accounting for the variable number of methylation sites in a single gene.

Finally, we aligned all DNAm genes to genes in the CNV and RNAseq data, and further aligned associated samples. Genes lacking -omic data across any of the omics data types were subsequently removed. We compared the resulting DNAm gene-body methylation beta values obtained from utilizing the weighted average approach to both simple averaging and the pre-summary gene-body methylation beta values by inspecting the preservation of the distribution of beta values based on the approach. To additionally ensure that the approach does not lead genes towards statistical significance over effect size, we conducted significance testing on resulting gene-level methylation as well as prior site-level methylation.

To evaluate whether the resulting data from the multiomics integration maintained distinct molecular differences between groups, the results were assessed for significant differences between TWAs and TMAs. Finally, correlation analysis was performed using Pearson’s correlation coefficient, reporting the median and standard deviation (STD) of gene coefficients on the IM datasets to evaluate the similarity of information provided from the different omics data types.

To characterize the loss in gene coverage resulting from integrating the multiomics data to a gene-centric format, we calculated the number of genes found in each -omic in the aligned samples prior to multiomics integration, and the number of genes found in the post-integration dataset. To assess functional bias, we additionally calculated the number of genes lost according to their functional categories.

### Pathway analysis workflow in SOPA into SIMPA

To conduct pathway analysis using single-omics in single samples, SOPA utilizes custom ranking metrics for RNAseq, CNV, and DNAm data, that compare each gene within a single sample to a range of controls. The custom ranking metrics were designed to assess the degree and direction of deviation of a single gene in a given sample from the majority of a reference population. As shown in Fig. 1, when inputting personalized gene ranking profiles for each sample in SOPA through the GSEA engine (in GSEApy v1.1.3 package [35]), the algorithm outputs normalized enrichment score (NES) for each pathway in a pathway library for every sample. In this research, we used the HALLMARK cancer pathways acquired from MSigDB (https://www.gsea-msigdb.org/) [36]. In SOPA, NES is calculated by permuting gene labels for each term in single samples over 1000 iterations, providing a corrected $P$-value indicating the likelihood of NES accuracy over chance. Due to the large number of tests conducted against pathways, multiple testing correction was done and BH–corrected $P<.05$ was considered statistically significant.

Results acquired from single -omic SOPA data analysis can be integrated downstream through SIMPA to gain more comprehensive insights into pathway activity. Here, SIMPA calculates a multiomics pathway enrichment score (MPES) to assess the degree and direction of the deviation of each pathway in a single sample from a reference population, using the single -omics’ NES values. Further details on deriving MPES and assessing its robustness are detailed in Supplementary Method 2. SIMPA further calculates the significance of pathway activity alteration by combining $P$-values obtained through SOPA analysis through the Stouffer’s method, which is appropriate when integrated -omics are assumed to have equal weights [37, 38]. To support the assumption of whether the single -omics data types have similar weights, we tested for correlations between the data types through Pearson’s correlation coefficient. An absolute median coefficient of $<0.39$ was considered indicative of a weak correlation [39] and would allow for Stouffer’s method to be utilized.

### Control–normalized gene ranking in RNAseq-based and DNAm-based SOPA

In group-based GSEA, minimum significant difference (MSD) is used to determine the smallest difference that can be considered statistically significant between two group means [40]. Here, we developed a customized MSD implementation to determine the smallest significant difference between a sample’s expression value and median of the control group’s expression, anchored by an implementation of a directional median absolute deviation. A thorough mathematical derivation of $\mathrm{MSD}_{D_{x,s}}$ is provided in Supplementary Method 3, and the final notation is summarized in equation 1. 


(1) \begin{align*}& \mathrm{MSD}_{D_{x,s}} = \frac{x_{s} - \tilde{x}}{\mathrm{MSD}_{x}^{\pm}}\end{align*}

where $MSD_{x}^{\pm }$ refers to directional MSD for gene x, $x_{s}$ for the expression of x in single sample s, and $\tilde{x}$ to the median expression of x in the control group (TWAs). $\mathrm{MSD}_{D_{x,s}}$ has several advantages over classical group-based MSD, as values above 1 or lower than −1 indicate significant deviations from TWA values. In addition to the interpretability of significance in the metric, it maintains the direction of alterations, as negative values represent downregulation, with positive values representing upregulation from the TWA value range. Since MSD is typically utilized for RNAseq data [40], we sought to develop a separate algorithm for DNAm data, based on the DNAm ranking algorithms using single sample ordinary least square (OLS) regression (details found in Supplementary Method 4). However, $\mathrm{MSD}_{D_{x,s}}$ showed superior performance to single sample OLS (Supplementary Fig. S1), and thus, $\mathrm{MSD}_{Dx,s}$ was used for DNAm data analysis in subsequent computational experiments.

### Adjusted weighting of gene-level copy numbers in CNV-based SOPA

An algorithm devised by Hsu *et al.* [41] named Gene Set analysis for Copy number Alteration (GSCA) was demonstrated to be capable of identifying CNA-driven biological pathways in breast cancer. Nonetheless, the GSCA implementation suffers from a number of major limitations. Firstly, it does not directly rank genes and instead focuses on identifying the number of genes altered in a gene set, and performs Fisher’s exact test to identify $P$-values. Secondly, GSCA lacks correction for multiple hypothesis testing, which would result in a high number of false positives when performing term enrichment.

To address GSCA’s limitations, we developed an algorithm that allows for the ranking of each gene according to the deviation of its CN from the control samples. The weighting method includes a condition to weight genes in a sample that have a neutral CN by calculating a baseline importance that adjusts to the STD of control values. The mathematical derivation is provided in Supplementary Method 5, and the resulting formula is shown in equation (2). 


(2) \begin{align*}& W_{\mathrm{adjusted}}(G_{x,s}) = \begin{cases} w(G_{x,s}), & \text{if } G_{x,s} \neq 2 \\ B_{x}, & \text{if } G_{x,s} = 2 \end{cases}\end{align*}

where $w(G_{x,s})$ refers to the weighted non-linear importance of gene x in sample s, in cases where $CN \neq 2$. While $B_{x}$ refers to baseline importance when $CN=2$. This method allows the extraction of genomic weights and maintains comparability between each sample and the controls, with the addition of allowing for the interpretation of directionality of gain/loss CNAs. Finally, as the metric is designed to be utilized in the GSEA algorithm, it inherently undergoes multiple testing correction.

### Comparison of SOPA and SIMPA against other methods

We considered several single sample pathway analysis tools [42] to compare to SOPA, and to ensure fair comparisons, we excluded tools that are designed for specific-omics, and tools that do not return results directly comparable to the standard GSEA algorithm. A description of the investigated single -omic tools for possible comparisons and the rationale for comparison inclusion is available in Supplementary Table S1. Here, we compare SOPA to ssGSEA and GSVA, due to their results being comparable to SOPA.

To assess the stability of the ranking metrics used in SOPA, we first calculated the metrics for all samples in the TCGA datasets (both TWAs and TMAs), establishing the “ground-truth” of the metrics. Then, we extracted random subsets of the control group (TWAs) and calculated the metrics again to compare to the “ground-truth” through correlation analysis. The random subsets were extracted at different sizes ($n=$ 3, 5, 10, 20, 50, 100, 200, 400, and full control group size) to assess the stability of the metrics at different control group sizes. The correlation analysis was performed using Spearman’s $\rho $ to account for the non-normal distribution of the metrics. When the correlation coefficient of $\rho> 0.9$ was reached at the smallest control group size, we considered the metrics to be stable at that threshold.

To validate the control-normalized ranking metrics employed in SOPA, we compared them to group-based ranking metrics. This included comparing $\mathrm{MSD}_{D_{x,s}}$ to group-based $\mathrm{MSD}$ for RNAseq data, as well as against group-based linear regression in DNAm data (methodology is available in Supplementary Method 6). Since there are no equivalent ranking metrics for CNV data, we compared CNV-based SOPA with GSCA, with details of comparisons between $W_{\mathrm{adjusted}}$ CN-weighting scheme provided in Supplementary Method 7.

We further aimed to assess the performance of the SOPA pipeline for RNAseq, CNV, and DNAm data on the TCGA dataset against ssGSEA, and GSVA (Supplementary Method 8) available in the GSEApy v1.1.3 package [35]. To ensure the performance of SOPA is not limited to the TCGA dataset, we conducted separate analyses on the external GEO datasets described in Table 1 with established biological patterns. The GEO datasets were chosen as each had two cohorts of patients, implementing a case-control/baseline-treatment design. Thus, our comparisons of the tools assessed the group-based patterns based on single sample analysis, to highlight the potential utility of SOPA in supervised contexts.

For IM single sample pathway analysis tools, a recent review [43] identified MOGSA and padma, as the only tools currently available for conducting such analyses, and recommended for the development of tools that build upon these approaches. Therefore, we evaluated SIMPA alongside MOGSA and padma on the IM dataset obtained from TCGA to identify pathway alterations between TWAs and TMAs. This approach was taken as to illustrate use-cases where predefined controls are available, where the inherent advantages of supervised analysis through SIMPA can be highlighted against unsupervised methods. Here, the MOGSA v1.40.0 R package and padma v1.22.0 R package were employed on the integrated data using the parameters described in the original papers [5, 6]. To optimize the performance of both tools, we employed them on log-transformed RNAseq, CNV and DNAm data, as recommended in the original papers. We additionally optimized the performance of padma by utilizing its reference-normalization functions, utilizing the control samples as a reference population, and projecting TMAs onto the reference. Additionally, we utilized Gaussian mixture model (GMM) regression in the scikit-learn v1.5.1 python package [44] on quantile transformed data to evaluate the capability of SIMPA and other tools to allow for the identification of tumor subgroups based on their immune-metabolic pathway activities. In the case where GMM clustering identified two stable clusters or more in a single group of patients, we employed xCell [45] clustering on the raw data of samples to identify correlations between immune infiltration scores and a tool’s scores. Finally, we conducted survival analysis using Kaplan-Meier curves and the log-rank test in the lifelines package [19] on the clusters, to confirm the clinical relevance of the clustering results.

### Statistical analysis

To calculate correlations, Pearson’s R or Spearman’s $\rho $ was utilized depending on whether the data was normally or non-normally distributed using the scipy v1.14.0 package [22]. We conducted analyses on the external GEO datasets using SOPA, ssGSEA, and GSVA, implementing the Mann Whitney U two-tailed test on the enrichment scores obtained with the three tools comparing two cohorts to highlight inter-tool differences. We chose to conduct the Mann Whitney U test as it allows us to assess whether the results obtained from each tool by comparing single samples to their controls, maintaining group-wide statistical significance, signifying the performance of each tool [46]. The significance threshold was set at $P<.05$ after BH correction for multiple testing. Similar approach was used when comparing SIMPA to MOGSA and padma. A complementary analysis was conducted to assess the difference in effect size observed between single -omic tools. We calculated Cliff’s Delta, and took the median Cliff’s Delta for all pathways. An absolute cliff’s delta of $<0.147$ was considered a negligible effect size, $<0.33$ a small effect size, $<0.474$ a medium effect size, and equal to or above 0.474 a large effect size, as per Romano *et al.* [47]. 95% confidence intervals for Cliff’s delta were calculated through bootstrapping with 1000 iterations. To report confidence intervals for all pathways, we provide minimum and maximum values to illustrate the range of the confidence intervals. To calculate an immune or a metabolic MPES score, we referred to the original HALLMARK pathways paper [36] and averaged immune MPES and metabolic MPES for all immune and metabolic pathways, respectively. Immune and metabolic gene set score (GSS) obtained by MOGSA was calculated similarly. For padma, we averaged the pathway deviation scores for the immune and metabolic pathways to obtain immune and metabolic pathway deviation scores. We calculated the immune infiltration score using the risk score formula by Luo *et al.* [48] MOGSA and padma implementations were conducted in R version 4.5.1 within a local Jupyter notebook environment. Calculation of immune cell composition was done through the xCell web application (https://comphealth.ucsf.edu/app/xcell). All other analyses were conducted in Python v3.11.9 within a Jupyter Anaconda environment. The python and R packages used in this article, are shown in Supplementary Tables S2 and S3, while the computational specifications are provided in Supplementary Table S4.

## Results and discussion

### Neoplastic tumors have complex multiomics, challenging standard analysis tools

**Figure 2:**
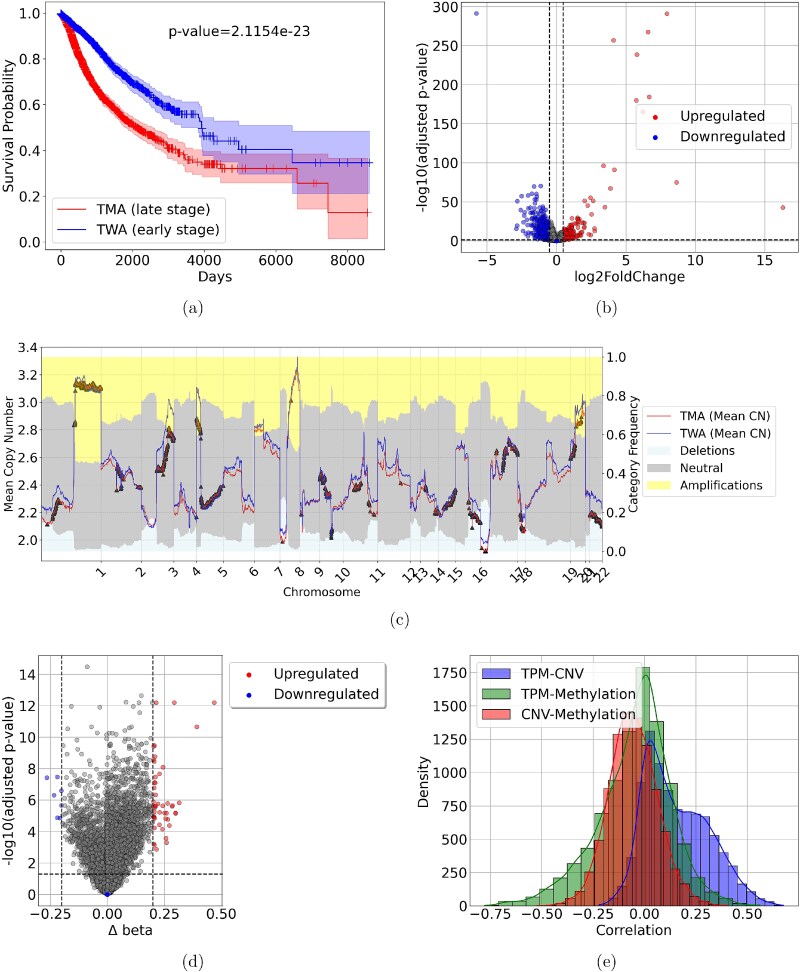
Analysis of survival profiles and molecular characteristics of the TCGA cohort. (a) Survival differences between early stage (TWA), and late stage cancers (TMA) ($n =$ 3186). Around 260 samples had missing overall survival data and were excluded. (b) Volcano plot illustrating differentially expressed genes, around 12.6% of the genes within the dataset were differentially expressed. (c) CNV data analysis. Background hue shows the frequency of mutations in TMAs (data points on the left y-axis). The red lines show the mean copy number in TMAs, and triangles show significant frequency of mutations in genes if the frequency of the mutations in the respective gene is significant compared to TWAs (blue line). (d) Differentially methylated genes constituted 5.3% of all genes within the dataset. (e) Distribution and density of Pearson’s correlation coefficients between the -omics showing generally low correlations across most genes.

To ensure that the TCGA samples were suitable for the analyses, we analyzed the survival and multiomics profiles for patients in both early stage (TWA) and late stage (TMA) cancer groups. 3186 out of 3446 patients had available survival data, we found clinically significant differences between the two groups (Fig. 2a, Supplementary Table S5). TMAs had distinctly worse survival (average days to death $766.6 \pm 788.9$) than TWAs ($1064.3 \pm 957.2$) at $P<.00005$. Assessing cancer subgroup survival (Supplementary Fig. S2) showed that the results were not skewed by a single cancer type. The survival discrepancy establishes that the TWA-TMA study design allows for the investigation of clinically meaningful differences that are involved in metastatic progression.

We further investigated whether there were molecular differences that reflected the survival disparity in both groups. Firstly, we used the largest possible single-omic cohorts, which spanned transcriptomics ($n=$ 3009), genomics ($n=$ 2514), and methylomics ($n=$ 2096 after excluding Epic platform data $n=$ 246). Here, TMAs consistently showed differentially altered genes compared to TWAs (Supplementary Fig. S3). Afterwards, we established a cohort of 1968 samples with integrated and aligned multiomics filtering for samples whose data was available (clinical descriptions available in Supplementary Dataset 1, and summary statistics are shown in Table 2).

**Table 2:** Demographics and clinical characteristics by cancer group.

**Cancer groups**	**Overall**			**TMA**			**TWA**		
	**Total**	**Males**	**Females**	**Total**	**Males**	**Females**	**Total**	**Males**	**Females**
	($n=1968$)	($n=681$)	($n=1287$)	($n=1089$)	($n=343$)	($n=746$)	($n=879$)	($n=338$)	($n=541$)
**Age** (median)	60	64	57.5	59	63	56	62	67	59
**STD**	14.26	13.11	14.48	14.71	13.72	14.91	13.51	12.28	13.79
**Cancer type**									
Breast	668	8	660	415	6	409	253	2	251
Lung	538	329	209	236	143	93	302	186	116
Thyroid	330	100	230	203	68	135	127	32	95
Colon	205	110	95	95	49	46	110	61	49
Pancreas	118	62	56	101	51	50	17	11	6
Kidney	109	72	37	39	26	13	70	46	24
**Cancer stage**									
I (I, IA, IB)	573	216	357	118	31	87	455	185	270
II (II, IIA, IIB, IIC)	782	252	530	426	132	294	356	120	236
III (III, IIIA, IIIB, IIIC)	475	148	327	416	118	298	59	30	29
IV (IV, IVA, IVB, IVC)	118	59	59	115	59	56	3	0	3
X	2	0	2	1	0	1	0	0	0

Importantly, the multiomics differences between the TMA and TWA groups were maintained after the alignment of datasets. Transcriptomics analysis showed a number of differentially upregulated and downregulated genes (Fig. 2b). Genomic mutation assessment of copy number frequency showed that TMAs had frequently higher deletions compared to TWAs across a large number of genes (Fig. 2c). The CNV dataset consisted of$\sim $20% significant TMA-associated mutations, including deletions, amplifications, and neutral CN. Differential methylation analysis had similarly consistent results, showing differentially methylated genes across TMAs and TWAs (Fig. 2d). These results suggest that there are multiomics-driven mechanisms that may be involved in tumor progression.

To assess whether each of the -omics analyzed provide different insights, we evaluated multiomics correlations in the aligned datasets. The datasets analyzed for correlations consisted of the integrated and aligned multiomics dataset of 1968 patient samples (Fig. 2e). Results showed that median Pearson’s correlation coefficients between TPM and CNV data were slightly positive 0.15 ($\pm $ 0.156), while TPM and DNAm beta values had low negative correlations with the median being −0.047 ($\pm $ 0.179), as for CNVs and DNAm, the median similarly was weakly negative with a mean of −0.055 ($\pm $ 0.11). Low correlations indicate that the data lacks redundancy and offers different insights. In addition, we found that our weighted averaging approach preserved the distribution of gene-body methylation, whereas the simple mean caused significant alteration (Supplementary Fig. S4).

In summary, the relatively low correlations between the -omics, and the presence of differentially altered genes in our TWA-TMA samples, indicate that each of the -omics contributes unique insights into -omic specific mechanisms significantly different in the two cohorts. Thus, these results provide an indication for combining $P$-values in SIMPA through the Stouffer’s method. Moreover, the significantly different alterations provide evidence that the TWA-TMA study design is appropriate to conduct analyses investigating molecular drivers of metastatic progression. Although we obtained gene-wide methylation values through $P$-value weighted averaging of methylation sites, the percentage of significantly differentially methylated genes was relatively similar to the pre-integration dataset, indicating that this approach did not bias the genes towards statistical significance.

### Control-normalization in SOPA is sensitive to differential pathway activity

After establishing the complexity and suitability of the TCGA dataset for subsequent analyses, and the identification of genes involved in the metastatic progression, we sought to first assess the performance of the ranking metrics used in our single -omic gene set analysis tool (SOPA) in preserving group-based trends and the capability to detect single gene perturbations in the TCGA dataset.

We compared the CNV ranking metric for single samples to the ranking metric used in GSCA. Results showed that CNV-based SOPA was capable of identifying more significantly enriched gene sets in more samples (as described in Supplementary Fig. S5). Additionally, we found that $\mathrm{MSD}_{D_{x,s}}$ was accurate in identifying single sample single-gene perturbations, while simultaneously preserving group-based trends in both RNAseq and DNAm datasets (results shown in Supplementary Figs S6 and S7 respectively). Taken together, the results show that the single sample ranking metrics preserve group-based trends. Simulations conducted to assess the stability of the metrics showed that the metrics were stable at a control group size of 10 samples (Supplementary Fig. S8).

After assessing the ranking metrics, we confirmed the improved differential pathway detection of SOPA over ssGSEA and GSVA in the TCGA dataset across RNAseq, CNV, and DNAm data (Supplementary Fig. S9). Across the two groups, we expect that the effect sizes of the differences between TWAs and TMAs would be significantly small if not negligible, as the dataset is highly heterogeneous and includes multiple cancer types, stages, and subtypes. Using Cliff’s delta, we found that the effect sizes of the differences between TWAs and TMAs across all tools were uniformly negligible or small (Supplementary Table S6), which is consistent with the heterogeneity of the dataset. Since Cliff’s delta was not sufficient in discriminating between the tools, we examined the number of significant pathway alterations between the two groups. Additionally, we calculated the number of significant pathway enrichments in each sample across the three -omics data types at $FDR<0.05$ (Supplementary Fig. S10).

It is clear that the ranking metrics implemented in SOPA affected enrichment score distributions, allowing the tool improved capability to identify differentially altered pathways in single samples, thereby maintaining group-specific patterns during group-based comparisons of results obtained using the mentioned tools (as shown in Supplementary Fig. S11). These results further support that the effect size differences between the two groups regardless of the magnitude, are preserved through SOPA’s inherent supervised approach. Therefore, SOPA is suitable for analyzing heterogeneous datasets in supervised contexts where pre-defined control groups are availabe.

**Figure 3:**
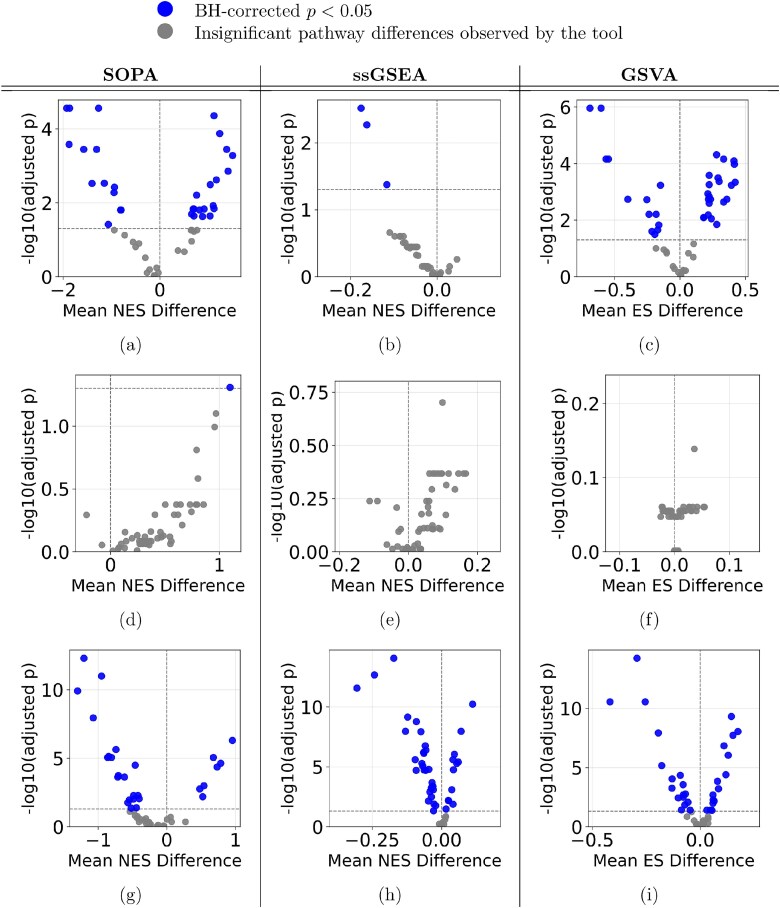
Comparison between SOPA, ssGSEA, and GSVA on external datasets from GEO for RNAseq, CNV, and DNAm data. (a–c) SOPA identified 31 pathways as significantly differentiated between treated samples at 3 weeks and at baseline from the GSE165252 RNAseq dataset, while ssGSEA was only able to identify 3 differentially altered pathway and GSVA 35 ($n=77$). (d–f) Of all tools, SOPA was capable of identifying one significantly differentiated pathway between primary bladder cancer tissue and metastatic tissue in the GSE39280 CNV dataset ($n=34$). (g–i) In the DNAm GSE207460 dataset, SOPA identified 27 significantly differentiated pathways. Whereas ssGSEA identified 47, and GSVA 32 ($n=154$).

To confirm the functionality of SOPA on RNAseq data, we used an external RNAseq dataset from GEO. The RNAseq external dataset included 77 esophageal carcinoma patients’ data treated with a combination of chemoradiotherapy with atezolizumab, assessed at baseline and at 3 weeks of treatment [49]. Through SOPA, we identified a global trend using the Mann Whitney U test to compare matched patients at baseline and at 3 weeks. SOPA showed that the treatment combination significantly altered a large number of HALLMARK terms (Fig. 3a), exceeding ssGSEA (Fig. 3b) but similar to GSVA (Fig. 3c). On the RNAseq dataset, SOPA provides improved interpretability due to its control normalization while preserving group-based patterns.

Next, we similarly assessed differences observed through CNV-based SOPA and ssGSEA and GSVA on an external dataset to confirm the internal TCGA dataset results. The CNV dataset involved analyses by Riester *et al.* [50] that identified copy number gains in metastatic urothelial tumors when compared to primary tumors. In this dataset, SOPA was the only tool to identify significant differentiated pathways between the two groups (Fig. 3d). In contrast, both ssGSEA (Fig. 3e) and GSVA (Fig. 3f) failed to identify differential patterns between the two groups. However, we acknowledge that the CNV dataset shows limited results, likely due to the sample size, as the performance in the TCGA dataset was different compared to GEO. Results obtained through CNV-based SOPA showed that it was capable of detecting significant enrichment across different samples due to the custom metric for the data type in comparison to other tools.

Afterwards, we sought to assess the performance of DNAm-based SOPA on an external dataset compared to ssGSEA and GSVA. The DNAm dataset GSE207460 by Fleischer et, al. [51] included methylation analysis on tumor biopsies before, during, and after treatment of triple negative breast cancer patients with either neoadjuvant chemotherapy alone or with bevacizumab to develop a predictive treatment signature. Here, SOPA similarly provides insights by identifying large single-sample perturbations while preserving group-based differential pathway alterations (Fig. 3g). SOPA shows comparable performance to both ssGSEA (Fig. 3h) and GSVA (Fig. 3i), as it found significant group-based patterns at the adjusted $P$-value threshold, while simultaneously providing control-normalization and per-sample false-positive control.

Taken together, our results indicate that SOPA provides improved interpretability of pathway alterations in single samples while preserving group-based trends, compared to ssGSEA and GSVA. Therefore, SOPA provides valuable insights in contexts where differential patterns in single samples in relation to a control group are of interest, with the added benefit of maintaining group-specific trends. Mainly, SOPA allows for the integration of single -omics results into SIMPA, which combines the results of different -omics to provide comprehensive insights into the deviation of single samples from a control group. While the TCGA dataset contains imputed data that may bias results, the external datasets serve as supporting evidence that the SOPA algorithm is functional and provides comprehensive insights in datasets that have not been exposed to imputation.

### SIMPA dissects immune–metabolic dysregulation in tumor subpopulations

**Figure 4:**
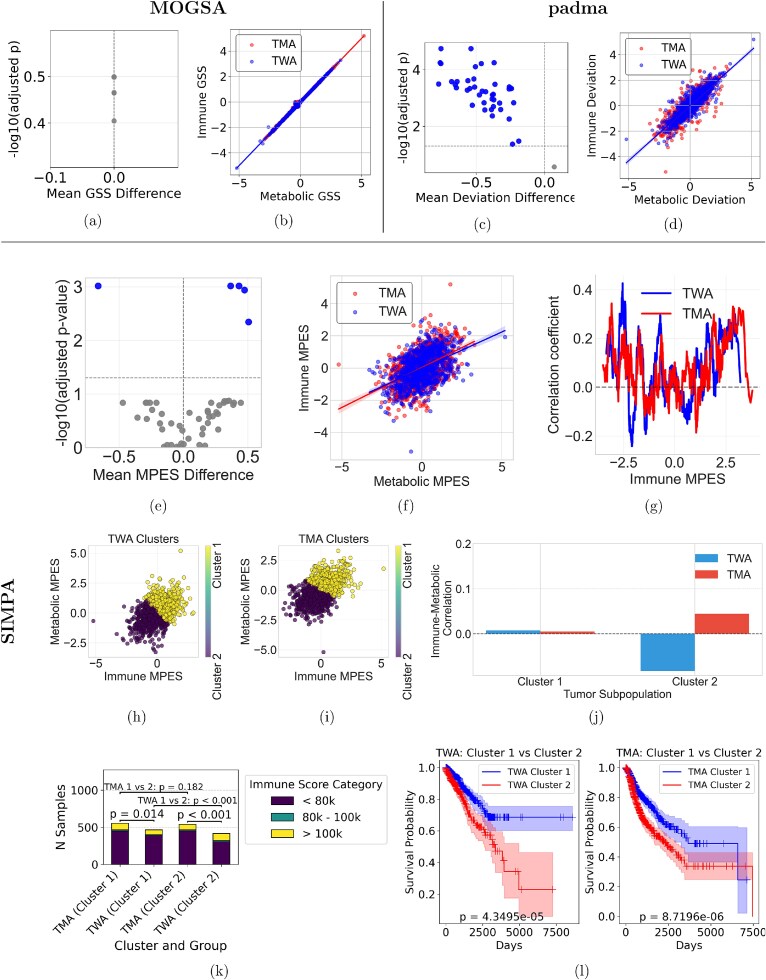
Investigation of the TCGA early stage (TWA) and late stage (TMA) cancer group datasets through IM pathway analysis using MOGSA and SIMPA ($n=1968$). (a) MOGSA was unable to detect any significantly different patterns between TWAs and TMAs. (b) GSS obtained by MOGSA did not show any immune and metabolic differences for TWAs and TMAs. (c) Deviation scores obtained through padma show that almost all pathways were significantly different between TWAs and TMAs, due to the nature of magnitude-based deviation scores and the heterogeneity of the dataset. (d) padma identified less linear relationships between the immune and metabolic pathways compared to MOGSA. (e) In contrast, SIMPA identifies significantly different patterns between terms across TWAs and TMAs. (f) Immune and metabolic MPES shape a non-linear cluster confirming the heterogeneity of relationships between the pathways. (g) Immune MPES has different correlation coefficients with metabolic MPES throughout the population. (h) and (i) GMM shows clear separation between the two cancer groups into cluster 1 (low-activity) and cluster 2 (high-activity). (j) Correlations between immune and metabolic pathways are different between clusters 1 and 2. (k) xCell predicts that clusters are biologically distinct based on RNAseq data, having significantly different immune cell infiltration scores across cancer groups. (l) Kaplan-Meier plots suggesting significantly worse survival for the TMA cluster 1 compared to TWA cluster 1 and for TMA cluster 2 compared to the TWA cluster 2.

After evaluating the performance of SOPA in single -omics, we sought to highlight the utility of SIMPA’s supervised methodology in our IM TCGA dataset, compared to both MOGSA and padma. In the TCGA dataset, MOGSA identified no significantly enriched pathways when utilizing the combined multiomics data (Supplementary Dataset 2). Moreover, the tool was unable to identify any significant differences between the early stage (TWA) and the late stage (TMA) cancer groups (as shown in Fig. 4a) or variance in immune-metabolic interactions (Fig. 4b). In contrast, padma does not output $P$-values, and therefore we were unable to assess the significance of the results obtained by padma. However, when comparing the mean deviation scores of pathways between the two groups, almost all pathways reached statistical significance (Fig. 4c), although correlation were more aberrant (Fig. 4d). Further analyses (Supplementary Fig. S12) highlight the need for supervised IM single sample pathway analysis approaches.

Confirming our earlier results, MOGSA identified low correlations between the -omics (as shown in Supplementary Table S7) and was therefore unable to provide meaningful insights into the dataset and metastatic progression-related pathways as it attributed the highest importance exclusively to the RNAseq -omic (Supplementary Fig. S13).

In contrast, SIMPA identified 17 492 significantly differentiated pathways in 1944 patients, with significant differences among cancer patient groups (Fig. 4e). When investigating the differences between the immune and metabolic activity of the TWA and TMA groups, SIMPA showed variation between the different cancer stages (Fig. 4f). Here, SIMPA identified that the correlation between immune and metabolic pathways was unstable and varied largely throughout metastatic progression across both early- and late-stage cancer groups (Fig. 4g). Therefore, these results highlight the importance of supervised control-normalization in dissecting complex datasets, as SIMPA allowed for the identification of single-sample perturbations while preserving group-based differences.

To showcase the utility of SIMPA in dissecting tumor heterogeneity, we sought to investigate immune-metabolic dysregulation through GMM clustering of SIMPA results. Here, we identified two distinct subpopulations in both TWAs and TMAs (Fig. 4h, i). The two clusters were characterized by their immune and metabolic activity, with cluster 1 (low-activity cluster) consisting of samples with low immune and metabolic activity, while cluster 2 (high-activity cluster) consisted of high immune and metabolic pathway activity. The clusters showed an immune-metabolic shift throughout metastatic progression (Fig. 4j), with correlations between immune and metabolic pathways increasing in cluster 2, across TWAs and TMAs. Therefore, these results indicate potential immune-metabolic coupling and decoupling in specific patients depending on the specific immune-metabolic profiles within our dataset, which were not identified by unsupervised methods (Supplementary Fig. S10).

Importantly, the immune infiltration score analysis of the raw data provided further supporting evidence for potential the biological effects of our clustering analysis (Fig. 4k). Notably, we found that the immune scores of the high-activity cluster were significantly elevated compared to the low-activity cluster for TWAs ($P<.001$) but not for TMAs. Survival analysis of the patients’ clinical profiles comparing cluster-specific survival further confirmed the clinical significance of our analysis and SIMPA. Here, cluster 1 survival of the early stage (TWA) group was significantly higher than that of cluster 2 ($P<.00005$) as shown in Fig. 4l. Similarly, cluster 2 in the late stage cancer group had significantly worse survival compared to cluster 1 in TMAs ($P<.00005$).

Overall, our results highlight that in contexts where predefined control groups are available, with low inter-omic correlations, unsupervised methods such as MOGSA and padma may not be optimal in identifying single sample perturbations while preserving group-based trends. Importantly, MFA tools such as MOGSA can be impacted by the nature of discrete CNV data, regardless of transformation, which likely contributed to its decreased performance in our dataset. Although padma provides improved performance compared to MOGSA, the heterogeneity of our dataset caused the magnitude-based deviation scores to be significantly different across all pathways between the two groups. While unsupervised methods can be more advantageous in contexts where different -omics are available (such as miRNAseq), or when control groups are not available, our results suggest that a supervised approach such as SIMPA is more effective in dissecting heterogeneous, controlled datasets with low inter-omic correlations. Here, SIMPA identifies differential pathway activation in single samples and provides insights into group-trends in low-correlating IM datasets composed of RNAseq, CNV, and DNAm data. In the TCGA dataset, SIMPA identifies unique immune-metabolic dynamics that couple or decouple according to the specific sample multiomics profile. The clinical significance of SIMPA analyses is supported by the differences in immune cell infiltration of tumor subgroups, and the survival discrepancy between the high-activity and low-activity clusters. However, we acknowledge that the immune cell infiltration scores were calculated using transcriptomics data, which requires further validation to confirm these results. Additionally, we confirmed that MPES aggregation maintains statistical calibration during the integration of single -omic scores (Supplementary Fig. S14).

The limitations within our study require further discussion. Gene-centric multiomics integration has been suggested to provide increased biological interpretability and condensed gene numbers [6, 52]. However, this integration approach faces the challenge where all assayed biomolecules need to be shared across all -omics [53, 54]. This is particularly relevant in our dataset, where our analyses were limited to the genes present in the least comprehensive assay, which was the gene-body methylation dataset with 11 369 genes. It is important to note that methylation arrays preferentially target protein-coding genes, which has been reported in the literature to be up to 75% of the probes depending on the platform [55]. Consistent with this design bias, we found that the data loss reflected the methylation array platform design (Supplementary Table S8), where approximately 82% of the 11 369 genes were protein-coding genes, leading to a final integrated dataset with around 95% of protein-coding genes retained from the methylation dataset. We acknowledge that the data loss may lead to biological bias due to the exclusion of non-coding genes that may carry independent biological signals, which is a limitation of this approach. Nonetheless, the clinical correlations of the analyses conducted on the integrated dataset suggest that the results remain meaningful. Future research would benefit from the use of more comprehensive methylation assays, such as the methylation EPIC array, which provide higher gene coverage and reduce the data loss in gene-centric multiomics integration. It is worth noting that while the imputation methods used have been proven to be effective in the literature [9, 10], imputation could introduce bias. Additionally, due to the lack of true values for the missing data, it is difficult to assess the impact of imputation on the results. Thus, it is important to further validate our design in future studies with more complete datasets. In gene-centric multiomics integration, the strict requirement for the presence of the same gene across all -omics imposes a necessity to perform imputation to reduce data loss, which has been the main approach in padma [6]. Moreover, the gene-level methylation aggregation strategy requires further discussion. Gene-level methylation summaries in the literature face the challenge of mapping promoter sites to a single gene, where recent research suggested that the weight of the methylation site would be divided by the number of genes it is mapped to [26]. One limitation in the software presented in [26], is that GOmeth does not allow for the extraction of gene-level methylation summaries, and instead focuses on gene set analysis. On the other hand, padma gene-level aggregation focused on utilizing the single most variable methylation site as representative gene-level methylation summary, which discards a large amount of information regarding gene-level methylation. Since it is beyond the scope of this paper to conduct a comprehensive comparison of gene-level methylation aggregation strategies, we opted for a weighted average approach that prioritizes significantly methylated sites while taking non-significantly methylated sites into account [56]. While our method of calculating gene-level methylation summaries was conducted on gene-body methylation sites, it is important to note that promoter methylation sites are also important in gene regulation. Future research should investigate the feasibility of developing more complex gene-level methylation aggregation strategies that take promoter methylation sites into account to provide more comprehensive gene-level methylation summaries.

It is worth noting that most gene sets available in the literature are based on transcriptomics data annotations, future research should focus on developing gene sets based on other -omics data types, to improve the interpretability of results obtained from multiomics analyses. Due to the nature of gene set annotations and our observational analyses, as well as sample heterogeneity, the findings regarding immune-metabolic dysregulation using IM cannot be generalized without further clinical validation. Although there is an available meta-analysis comparing ranking metrics for GSEA, the review only utilized RNAseq data, which leaves room for further investigations into optimal ranking metrics for other -omics [40]. Moreover, as our pipeline is computationally expensive due to performing calculations on every single sample, our analysis was limited to HALLMARK terms, which is important to address in future research through the exploration of other pathway libraries, such as GO Biological Processes. Although we used external datasets to evaluate our pipeline, future research is required to confirm our results both in vitro and in clinical studies. Particularly, patients obtained through TCGA all received treatment at different stages of disease, requiring further investigations in vitro regarding the nature of immune-metabolic dysregulation in metastatic progression.

## Conclusion

In conclusion, we have shown that the TNM staging-based study design provides a valuable opportunity for future research to dissect metastatic progression and its variability across different solid tumor types. Due to the methodology implemented in SOPA, we showed that the framework was able to determine a number of consistently altered pathways in late TNM-stage compared to early stage cancer groups. Here, SOPA was able to detect more significantly differentially altered pathways compared to other tools such as ssGSEA and GSVA in both the TCGA, and external GEO single -omic datasets. Confirming our analysis of the low correlations between the single -omics in the TCGA dataset, MOGSA similarly detected low associations between the -omics and was unable to identify any significant pathway alterations in single samples using the IM consisting of RNAseq, CNVs, and DNAm data. Padma showed improved performance compared to MOGSA, but the heterogeneity of the dataset in a supervised context caused padma to have decreased performance. Here, SIMPA was able to determine a large number of differentially altered pathways on the same dataset, which had significant correlations with the cellular composition of the tumors predicted by using xCell data. Additionally, based on the immune-metabolic hallmark pathways, SIMPA showed patient clusters within both early and late stage tumors, which had significantly different survival outcomes. Both SOPA and SIMPA offer valuable insights to allow for improved detection and interpretability of single-sample alterations in supervised analysis contexts involving disease heterogeneity, and treatment-response assessment of single samples. Further research is necessary to investigate the immune-metabolic observations predicted by SIMPA.

**Key Points**

- Separating tumors into early stage and late stage groups based on the nodal involvement through TNM staging allows for the investigation of clinically relevant differences in metastatic progression. This case-control study design is marked by significant survival and molecular differences between the early and late stage tumor groups.
- Control-normalization of gene ranking metrics in single sample single-omic pathway analysis (SOPA) allows for the preservation of group-based trends in single sample analyses. The outcomes of this methodology allows for the investigation of tumor heterogeneity more effectively compared to unsupervised standard tools.
- The single sample integrated multiomics pathway analysis (SIMPA) provides more nuanced insights into pathways regulating tumor progression compared to unsupervised tools such as multiomics gene set analysis (MOGSA) and PAthway Deviation scores using Multiple Factor Analysis (padma) in supervised analysis contexts. SIMPA suggests that potential immune-metabolic dysregulation can be implicated in metastatic progression across different types of solid cancers, marked by variable immune infiltration and clinically significant survival differences between tumor subgroups.
- Tumor heterogeneity can be effectively addressed through supervised single sample pathway analysis through SOPA and SIMPA through the comparison of cases to predefined controls, allowing for the identification of personalized pathway perturbations in single samples.
- SOPA and SIMPA can be used through the publicly available python package at https://github.com/hasanalsharoh/SIMPApy/.

## Supplementary Material

Supplementary_Dataset_1_Details_of_study_cohort_bbag338

Supplementary_Dataset_2_MOGSA_results_bbag338

Supplementary_Information_bbag338

## Data Availability

All the TCGA curated raw data can be found in the Zenodo repository: https://doi.org/10.5281/zenodo.20067447.

## Code availability

The code produced in this article to develop and validate the ranking metrics, and the SOPA/SIMPA pipeline can be found and replicated in the GitHub repository: https://github.com/hasanalsharoh/SIMPA/. The python package (SIMPApy) implementing SOPA and SIMPA can be found at https://github.com/hasanalsharoh/SIMPApy/.
