# Supplementary material for: SOPA and SIMPA: normalized single-sample integrated multiomics pathway analysis of tumor heterogeneity in solid cancers
Source: Brief Bioinform. 2026 Jul 8;27(4):bbag338. doi: 10.1093/bib/bbag338 (PMC13345383; doi:10.1093/bib/bbag338)
Supplement: Supplementary_Information_bbag338 [file supplementary_information_bbag338.pdf]

Supplementary Materials for
SOPA and SIMPA: Normalized single sample integrated multiomics pathway analysis of tumor heterogeneity in solid cancers

Hasan Alsharoh[1,2,*], Abdulrahman Ismaiel[3], George A. Calin[4,5,6], Ovidiu Laurean Pop[1], Ioana Berindan-Neagoe[2,7,8,*], and Andreas Bender[9,2,10,11]

[1]Doctoral School of Biomedical Sciences, Faculty of Medicine, University of Oradea, Oradea, Romania
[2]Department of Genomics, MEDFUTURE Institute for Biomedical Research, Iuliu Hațieganu University of Medicine and Pharmacy, 400337 Cluj-Napoca, Romania
[3]2nd Department of Internal Medicine, Iuliu Hațieganu University of Medicine and Pharmacy, 400006 Cluj-Napoca, Romania
[4]Department of Cancer Biology, The University of Texas MD Anderson Cancer Center, 1515 Holcombe Boulevard, Unit 422, Houston, TX 77030, USA
[5]Translational Molecular Pathology, MD Anderson Cancer Center, Texas State University, 1515 Holcombe Blvd, Houston, TX 77030, USA
[6]The RNA Interference and Non-coding RNA Center, MD Anderson Cancer Center, Texas State University, Houston, 1515 Holcombe Blvd, Houston, TX 77030, USA
[7]Doctoral School, Iuliu Hatieganu University of Medicine and Pharmacy, Cluj-Napoca, Romania
[8]Academy of Medical Sciences, Bucharest, Romania
[9]Department of Medicine and Center for Biotechnology, College of Medicine and Health Sciences, Khalifa University of Science and Technology, Abu Dhabi, United Arab Emirates
[10]STAR-UBB Institute, Babeş-Bolyai University, Cluj-Napoca, Romania
[11]Centre for Molecular Informatics, Department of Chemistry, University of Cambridge, Lensfield Road, Cambridge CB2 1EW, United Kingdom
[*]Corresponding author: Ioana Berindan-Neagoe, ioana.neagoe@umfcluj.ro

Contents

Supplementary Methods **4**
Supplementary Method 1. TWAs and TMAs study design according to TNM & FIGO staging . . . 4
Supplementary Method 2. P-value aggregation and MPES calculation . . . 5
Supplementary Method 3. $MSD_{D_{x,s}}$ . . . 7
Supplementary Method 4. DNAm single sample linear regression . . . 8
Supplementary Method 5. Non-linear weighting for CNVs . . . 9
Supplementary Method 6. RNAseq and DNAm ranking metrics validation . . . 10
Supplementary Method 7. CNV ranking metric comparisons to GSCA . . . 11
Supplementary Method 8. Evaluation of SOPA against ssGSEA, and GSVA . . . 12

Supplementary Figures **13**
Supplementary Figure S1. Single sample linear regression agreement with group-based linear regression . . . 13
Supplementary Figure S2. Cancer-subgroup survival analysis . . . 14
Supplementary Figure S3. Pre-integration molecular profiles of TWAs and TMAs 15
Supplementary Figure S4. Assessment of p-value weighted aggregation on DNAm data in the post-integration dataset . . . 17
Supplementary Figure S5. CNV-based SOPA compared to GSCA . . . 18
Supplementary Figure S6. Group-based MSD and $MSD_{D_{x,s}}$ . . . 20
Supplementary Figure S7. Group-based regression and $MSD_{D_{x,s}}$ . . . 21
Supplementary Figure S8. Stability of SOPA ranking metrics . . . 22
Supplementary Figure S9. SOPA, ssGSEA, and GSVA in RNAseq, CNV, and DNAm TCGA datasets . . . 23
Supplementary Figure S10. TCGA supplementary comparison of SOPA, ss-GSEA, and GSVA . . . 25
Supplementary Figure S11. Impact of control-normalization on NES in SOPA, compared to ssGSEA, and GSVA . . . 26
Supplementary Figure S12. Results of MOGSA and padma analyses in the IM TCGA dataset . . . 27
Supplementary Figure S13. MOGSA determines RNA to be the most important component of the multiomics through PCA . . . 28
Supplementary Figure S14. Within-omic aggregation does not introduce bias in MPES scores . . . 29

Supplementary Tables **30**
Supplementary Table S1. Potentially comparable tools to SOPA . . . 30
Supplementary Table S2. Python packages . . . 33
Supplementary Table S3. R packages . . . 34
Supplementary Table S4. Computer specifications . . . 35
Supplementary Table S5. TWA-TMA survival differences . . . 36
Supplementary Table S6. Effect size differences observed by SOPA, ssGSEA, and GSVA, on the TCGA dataset . . . 37
Supplementary Table S7. RV coefficients in MOGSA . . . 38
Supplementary Table S8. Data loss resulting from multiomics-based gene-centric integration . . . 39

Supplementary References **40**

Supplementary Methods

Supplementary Method 1. TWAs and TMAs study design according to TNM & FIGO staging

To group samples into the designated groups, cancer types were parsed to streamline sample grouping. Additionally, the available data was searched to identify present classifications available in the dataset for assigning TMA and TWA status to samples. We used the 7th AJCC cancer staging manual as reference to develop the group-design [1]. We used custom python scripts to filter cancers according to different conditions, depending on cancer types present in the clinical information dataset retrieved from TCGA. For breast, lung, colon, pancreas, kidney, and thyroid cancers, TWA status was assigned if the tumor had a TNM N classification of N0, or $N0(i-)$, in addition to having an M classification of M0, or $cM0(i+)$. Where the TNM N and M classification information was not available, American Joint Committee on Cancer (AJCC) staging was further used. This was the case in colon cancer samples, and to address this, samples having AJCC stage of Stage I, Stage II, Stage IIA, and Stage IIB were considered TWAs, due to their definitions as to not having nodal extensions. Finally, for ovarian cancer, information was only available for the FIGO stage, and Stage IC was the only available staging fulfilling the criteria of TWAs.

For TMAs, in terms of breast, lung, colon, pancreas, kidney, and thyroid cancers, TMA class was designated for tumors with N class N0 (i+), $N0(mol+)$, N1, N1a, N1b, N1c, N1mi, N2, N2a, N2b, N3, N3a, N3b, and N3c. When N classification was not available, or classification was NX, we further parsed M classification. M1, M1a, and M1b classified tumors were designated TMAs. When both of N or M classifications were not available for colon cancer tumors, AJCC pathologic staging was searched, and TMA class was assigned if a sample's stage was Stage III, Stage IIIA, Stage IIIB, Stage IIIC, Stage IV, or Stage IVA. As for ovarian cancer, TMA class was assigned if FIGO stage was Stage IIIC, Stage IIIB, Stage III, Stage IV, or Stage IIIA. Finally, if the data involved lymphoma samples, or solid cancers with unavailable N or M classification, or Nx and Mx classifications, the samples were thereof excluded.

Supplementary Method 2. P-value aggregation and MPES calculation

In MultiGSEA [2], a combined Z-score would be obtained and used to express the combined enrichment of a pathway from the p-values of single-omics, thus expressing the magnitude of significance of pathway enrichment. Nonetheless, combined Z-score does not account for leading edge genes and matched genes of every-omic, and does not account for the NES provided by the GSEA algorithm. Therefore, in our implementation, in addition to calculating a combined Z-score for single samples, we also sought to introduce a more comprehensive assessment of the total enrichment of a pathway based on the contribution of every single-omic analyzed in single samples. When considering the original GSEA paper [3], NES is extracted after performing a number of permutations of gene rankings, and an adjusted p-value is provided after multiple testing to indicate false positive NES. Based on these calculations, we extracted adjusted p-values (FDR) to account for significance of enrichment and probability, NES to indicate magnitude of pathway enrichment, and number of leading edge genes and number of matched genes of a sample within a gene set to identify a weighted-omic contribution score (WOCS) for each pathway for every single sample. After normalizing WOCS scores and accounting for standard deviations of WOCS across omics, a resulting multiomics-based pathway enrichment score (MPES) was obtained. The calculation was performed as follows:

Assume adjusted p-value (FDR) in a single sample (s) indicates the likelihood of significance of enrichment magnitude (represented by NES) of a pathway (p) by a single-omic (o). Further we assume the leading edge contribution (L) of leading edge genes providing the highest contribution out of the matched genes in a pathway.

$$L = \frac{\text{number of leading edge genes}_{(o,p,s)}}{\text{number of matched genes}_{(o,p,s)}} \tag{1}$$

Here, assuming $WOCS_{o,p,s}$ as the single-omic contribution score for p in s, we reach the following equation:

$$WOCS_{o,p,s} = (1 - FDR_{o,p,s}) \times NES_{o,p,s} \times \log(1 + L_{o,p,s}) \tag{2}$$

Finally, we reach MPES through the sum of $WOCS_{o,p,s}$ values, and adjusting for the mean (μ) of $WOCS_{o,p,s}$ and standard deviation:

$$MPES_{p,s} = \frac{\sum_{o=1}^{\text{omics}} WOCS_{o,p,s} - \mu_{WOCS}}{\sigma_{WOCS_{p,s}}/\sqrt{k}} \tag{3}$$

Where $\sigma_{WOCS_{p,s}}$ across $k = 3$ omics is calculated as follows:

$$\sigma_{WOCS_{p,s}} = \sqrt{\frac{1}{k}\sum_{o=1}^{k}(WOCS_{o,p,s} - \mu_{WOCS})^2} \tag{4}$$

Overall, MPES provides a directional concordance score for combined multiomics enrichment of pathway activity, accounting for single-omic enrichment magnitude, significance, and leading edge gene contributions, and standardizing by deviation of WOCS values. Following the normalization of –omic scores, the implementation of MPES enables visualization of the combined direction and magnitude exerted by the combined multiomics on each pathway enriched in a single sample. Significance of each pathway

in every sample is determined through Stouffer's combined Z-score and its corresponding BH-corrected p-value. This implementation mirrors Stouffer's combined p-value calculation in multiGSEA [2], while providing additional metrics to assess various aspects of enrichment for single samples.

To further test whether the $WOCS_{o,p,s}$ aggregation leads to systematic biases or asymmetry in the distribution of MPES values, we implemented an aggregation-based null distribution. For each TMA sample's SOPA output for the three omics, we permuted pathway labels 10,000 times, and calculated the resulting MPES for each permutation. This allows for preserving each –omic's marginal distribution of values (NES, FDR, leading edge contributions) and removing cross-omic relationships for each pathway. The results of these permutations were then evaluated for symmetry, and median of the distribution of permuted MPES values.

Supplementary Method 3. $MSD_{D_{x,s}}$

We developed a customized directional MSD implementation to determine the smallest difference between a sample's expression value for a single gene and the median of the control group's expression. The mathematical notation is expressed as follows: Consider $\tilde{x}$ as the TWA (controls) group median for gene expression normalized counts for a gene x, if we are to determine MSD for x across accounting for control samples' variance:

$$\text{MSD}_x^{(\pm)} = z_{\alpha/2} \times \sigma_x^{(\pm)} \tag{5}$$

Where the $Z_{\alpha/2}$ represents the standard normal quantile defining the two-tailed critical value at α. Here, $\alpha = 0.05$, making $Z_{\alpha/2} \approx 1.96$, and thus providing a 95% confidence level for the assessment of significant deviations in gene expression values. which assesses the variance of values for a single gene within the control group. $\sigma_x^{(\pm)}$ represents the robust directional MAD for the positive and negative deviations, multiplied by the constant κ to make it consistent with the standard deviation for normal distributions. Here, dMAD allows for the splitting of deviations from the median, and the consideration of skewness in the data distribution. This implementation is additionally robust to asymmetric distributions around the median, and as follows:

$$\sigma_x^{(+)} = \kappa \times \text{MAD}_x^{(+)} \quad \sigma_x^{(-)} = \kappa \times \text{MAD}_x^{(-)}, \quad \kappa = \frac{1}{\Phi^{-1}(3/4)} \approx 1.4826 \tag{6}$$

The medians are calculated separately through:

$$\text{MAD}_x^{(+)} = \text{median}(\{x_i - \tilde{x} : x_i > \tilde{x}\}), \quad \text{MAD}_x^{(-)} = \text{median}(\{x_i - \tilde{x} : x_i < \tilde{x}\}) \tag{7}$$

Where x_i represents the gene expression value for a single sample within the control group. MSD was calculated for each gene and TWA gene expression values were considered as reference. Thus, MSD_x represented the least significant difference between each gene and its mean for the TWA group. For a gene to reach significance, the difference ($D_{x,s}$) between the gene (x) expression for a sample (s) and the mean of the control group must be assessed. $D_{x,s}$ defines the direction of expression (upregulation/downregulation) and the magnitude of deviation from the control median.

$$D_{x,s} = x_s - \tilde{x} \tag{8}$$

A sample's gene expression was considered to have statistical significance if the following condition is met:

$$|D_{x,s}| > MSD_x \tag{9}$$

Finally, $D_{x,s}$-directed MSD was used as a ranking statistic for SOPA:

$$MSD_{D_{x,s}}^{(\pm)} = \begin{cases} \frac{D_{x,s}}{\text{MSD}_x^{(+)}}, & \text{if } D_{x,s} \geq 0 \\ \frac{D_{x,s}}{\text{MSD}_x^{(-)}}, & \text{if } D_{x,s} < 0 \end{cases} \tag{10}$$

$MSD_{Dx,s}$ has several advantages over classical MSD, where if the value is above 1, it would indicate the presence of a significant alteration, with negative values implying downregulation, and positive values indicating upregulation. This effectively allows for the evaluation of significant deviations in expressions, and the direction of deviations.

Supplementary Method 4. DNAm single sample linear regression

To model linear regression to the use case in SOPA, several adjustments were made, and as follows: Assume $M_{x,s}$ as the methylation M-value for the xth gene in the sth sample, and C_x as a set of M-values for x. Consider a pseudoresponse variable (y), as a binary array of length $|C_x| + 1$, with zero values, representing controls, and +1 referring to the current sample. Linear regression was calculated through the OLS model in python. In this implementation, we would perform linear regression for each s sample to assess $\beta_0^{(s,x)}$ as intercept, $\beta_1^{(s,x)}$ as slope, ϵ as error, and p-value for x in s through:

$$y = \beta_0^{(x,s)} + \beta_1^{(x,s)} a + \epsilon \tag{11}$$

Finally, the ranking metric for DNA methylation using linear regression was calculated as follows:

$$\text{MethylRank}_{x,s} = \beta_1^{(x,s)} \times \text{p-value}_{\text{adjusted}} \tag{12}$$

Supplementary Method 5. Non-linear weighting for CNVs

The conditions implemented by this weighting scheme provide an indication to the deviation for a single gene from the variation of copy numbers in control samples and the rarity of the copy number, aligning with the goals of providing a controls-normalized ranking metric for CNV data in single samples, and as follows:

Assuming C to be the set of control samples, with the number of C being N, and assuming $G_{x,s}$ be the copy number of gene x in current sample s, we have the following contingency table:

$$\begin{array}{ccc} & G = G_{x,s} & G \neq G_{x,s} \\ \text{Sample } s & 1 & 0 \\ \text{Controls } C & k & N-k \end{array} \tag{13}$$

Where k represents the number of control samples who have copy numbers equal to current sample. Further, to calculate the odds ratio (OR) avoiding the potential division by zero, we additionally apply continuity correction of 0.5. Resulting with the equation below:

$$OR_{corrected} = \frac{(1+0.5)\times((N-k)+0.5)}{(0+0.5)\times(k+0.5)} = \frac{1.5\times(N-k+0.5)}{0.5\times(k+0.5)} \tag{14}$$

We further account for the standard deviation of the copy numbers of gene x in C ($\sigma_{x,C}$), adding a small constant (ϵ) to avoid division by zero for low variance genes. By additionally accounting for direction ($sgn(G_{x,s}-2)$) to evaluate whether the gene is considered a gain or a loss, we end with the following conclusion:

$$W_{adjusted}(G_{x,s}) = \begin{cases} w(G_{x,s}) = \frac{sgn(G_{x,s})\times log_{10}(OR_{corrected})}{\sigma_{x,C}+\epsilon}, & \text{if } G_{x,s} \neq 2 \\ B_x = \frac{2-\mu_{x,C}}{\sigma_{x,C}+\epsilon}, & \text{if } G_{x,s} = 2 \end{cases} \tag{15}$$

The adjusted weights would then be usable for SOPA downstream. This method allows the extraction of genomic weights and maintains comparability between each sample and the controls, with the addition of allowing for the interpretation of directionality of gain/loss CNAs. Overall, this implementation provides an alternative to GSCA, due to the single sample normalization to the control group, and the integration with multiple testing.

Supplementary Method 6. RNAseq and DNAm ranking metrics validation

In terms of RNAseq ranking metric validation, $MSD_{D_{x,s}}$ values were averaged and the difference was measured between the medians of TMAs and TWA (cases and controls, respectively). The resulting values for the single sample-based aggregate for each gene were compared to the resulting values of implementing the group-based classical MSD implementation in GSEA by assessing the correlation through the Spearman's Rank test.

As for the DNA methylation metric, we tried both single sample linear regression metric ($\text{MethylRank}_{x,s}$), and $MSD_{D_{x,s}}$ and assessed their concordance with classical linear regression. The $\text{MethylRank}_{x,s}$ metric used here implements adjusted p-values and regression slope, where the resulting value would report the direction of methylation, as well as statistical significance. $\text{MethylRank}_{x,s}$, when aggregated, would result in a loss of coherence of the values due to the statistical properties of these results. Therefore, to assess concordance between the methylation metric for single-sample based and group-based regression analyses, we conducted Spearman's Rank test on the mean of slopes for single samples and the slope for group-based regression for each gene. Lastly, the concordance assessment for $MSD_{D_{x,s}}$ aggregates (median differences) was done against slopes of group-based regression.

Supplementary Method 7. CNV ranking metric comparisons to GSCA

To validate the utility of $W_{adjusted}(G_{x,s})$, we conducted CNV-based SOPA on the TCGA CNV dataset and compared the similarity of results to those of a custom GSCA python script which implements multiple testing. Due to the inherent differences between the modalities, we assessed the differences in gene sets identified in the CNV dataset and single sample term enrichment. Similarity between identified gene sets and sample-specific enrichment was assessed through the Jaccard's Index (JI) in python using the formula:

$$\mathrm{JI}_{A,B} = \frac{\mid A \cap B \mid}{\mid A \cup B \mid} \tag{16}$$

Where JI for tool A (SOPA) and tool B (GSCA), equalled the absolute number of items (whether significantly enriched pathways or samples with significantly enriched pathways) in set A intersecting with set B, divided by the union of the twos sets.

Supplementary Method 8. Evaluation of SOPA against ssGSEA, and GSVA

To demonstrate the difference in results obtained through ssGSEA and SOPA, we performed ssGSEA on integrated TCGA and GEO datasets. To conduct ssGSEA [4], we used the ssgsea module in the GSEApy [5] package in python, and set the sample normalization method to "log rank", and the correlation normalization type to "Z-score". ssGSEA was utilized on each sample, and the results were further analyzed, and compared to SOPA results. For GSVA [6], standard implementation in the GSEApy package [5] was used, and results were compared to SOPA. SOPA enrichment of terms was considered significant when $FDR < 0.05$.

Supplementary Figures

Supplementary Figure S1. Single sample linear regression agreement with group-based linear regression

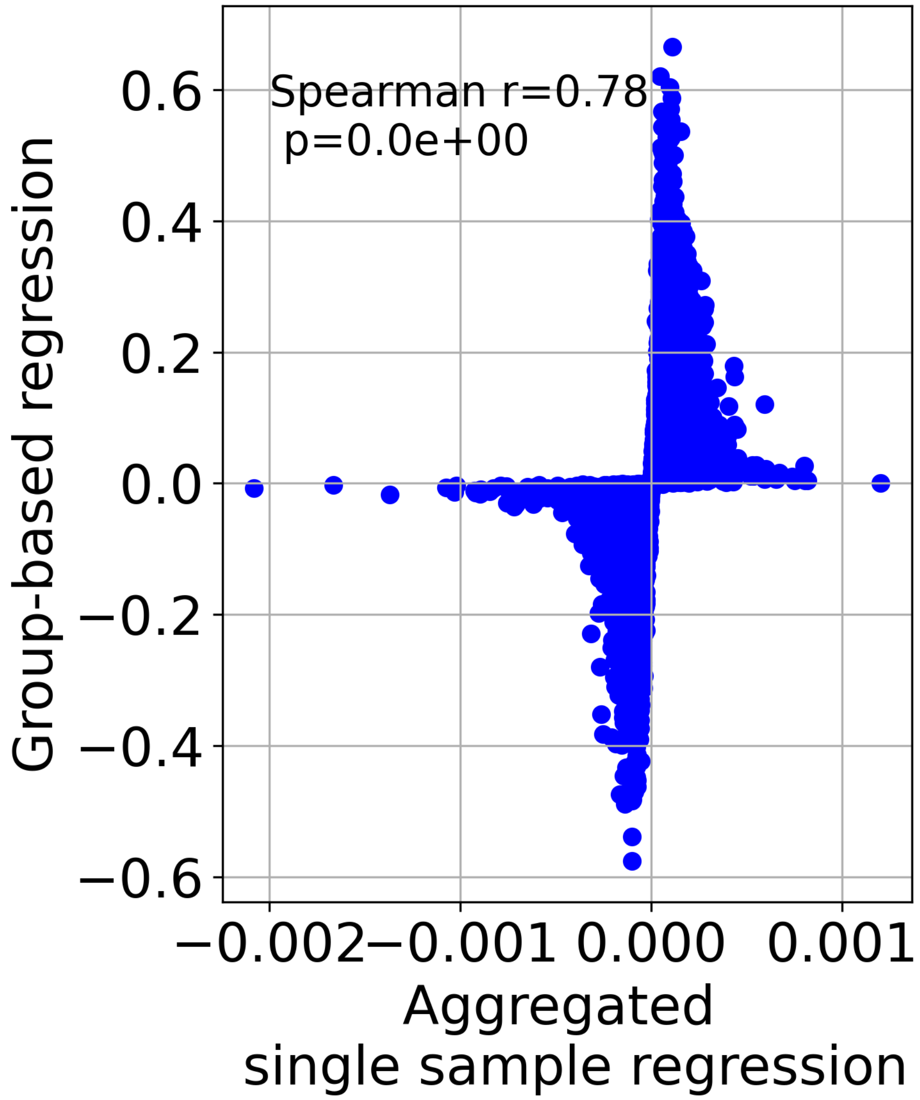

Figure S1: The concordance between the single sample regression, and group-based regression, Spearman's coefficient returned a test statistic of 0.78 ($p < 0.00005$), indicating high agreement between the results of the two methods.

Supplementary Figure S2. Cancer-subgroup survival analysis

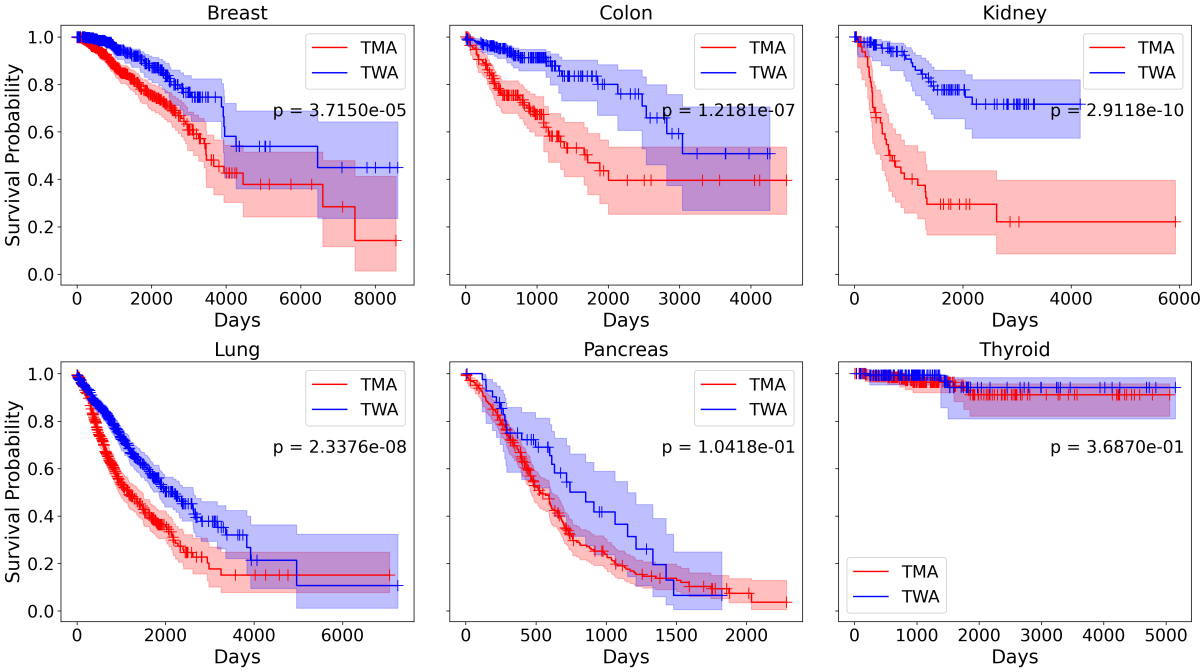

Figure S2: Kaplan-Meier survival analysis for cancer subgroups (n=3,186). For the censorship of survival data, samples without available cancer staging information, or samples without available overall survival time were dropped (n=260). Based on the results of the survival analysis, it is evident that the TMA-TWA study design carries considerable value in stratifying patient groups. Thus, investigating the molecular landscape of cancer using this design could reveal potentially relevant markers in metastatic progression. There were significant differences in survival between breast cancer TMA patients and TWA patients. (n=1,016) Colon cancer TMA and TWA groups showed large differences in survival post 2000 days. (n=435) Kidney tumor TMAs showed significant differences in survival compared to TWAs with kidney tumors. (n=150) Similarly, Kaplan-Meier analysis showed significant differences in survival between lung cancer TMAs and TWAs. (n=1,084) Pancreatic cancer TMA and TWA groups showed a small difference in survival, but the difference did not reach statistical significance. (n=311) Similarly, thyroid cancer patients designated TMAs, did not have significant differences in survival compared to TWAs. (n=384)

Supplementary Figure S3. Pre-integration molecular profiles of TWAs and TMAs

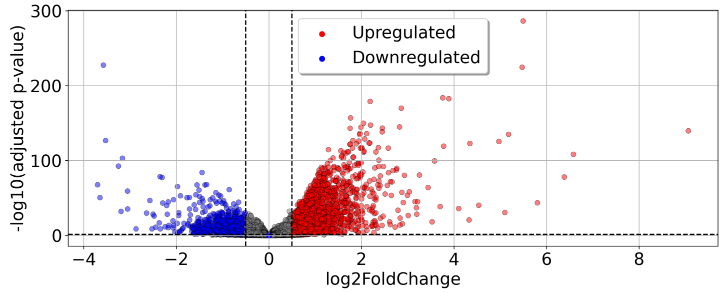

(a)

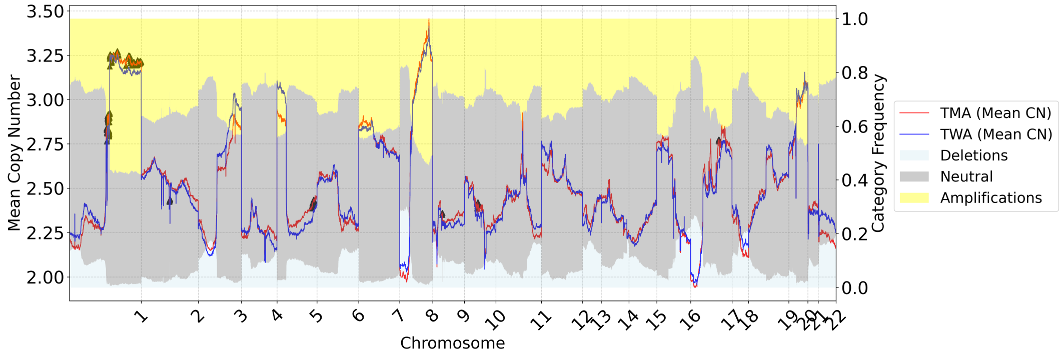

(b)

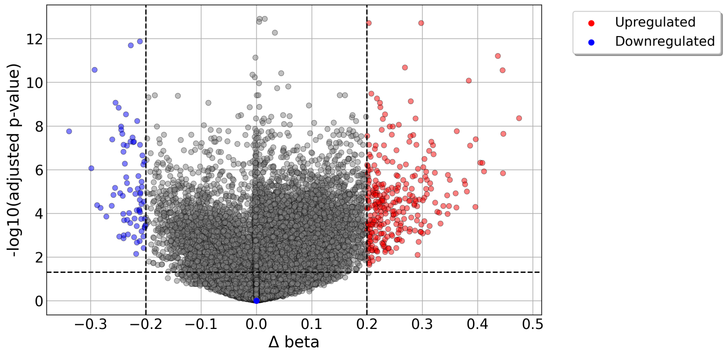

(c)

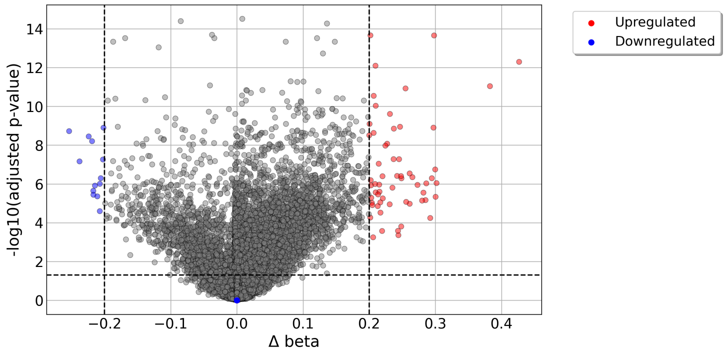

(d)

Figure S3: Multiomics differences between non-metastatic (TWAs) and potentially metastatic samples (TMAs). (Figure legend continues on next page)

Figure S3: (Figure legend continued from previous page) (a) Differentially dysregulated genes based on RNAseq data (n=3,009). This result indicates that there are molecular differences between TWAs and TMAs based on transcriptomics analysis. Differential expression analysis on RNAseq raw counts revealed that from the 48,107 analyzed genes, 4,295 genes were found to be significantly differentially expressed. The raw counts were transformed into normalized counts, and the results were visualized to illustrate the variation of gene expression across the analyzed population. Of the differentially expressed transcripts, 2,339 genes were upregulated and significantly differentially expressed, while 1,956 were significantly downregulated. (b)Analysis of CNV data for 2,514 patients of 55,820 genes revealed that 1,388 genes were significantly altered and involved in metastatic progression. We have found that the largest number of significantly altered genes was in chromosome 1 (1,150 genes), with 168 genes in chromosome 5. All significantly altered genes in TMAs were observed to contain deletions when compared to TWAs. (c) Out of the 91,230 gene-body methylation sites identified in 2,096 patients, 363 sites were significantly differentially methylated, indicating the presence of important methylation alterations in metastatic progression. Overall, 290 methylation sites were significantly hypermethylated, while 73 sites were significantly hypomethylated. (d) Methylation sites beta values were summarized into 11,369 genes. Of the genes, 73 showed significant differential methylation between the TWA and TMA groups. 60 genes were identified as differentially hypermethylated, and 13 genes as significantly hypomethylated.

Supplementary Figure S4. Assessment of p-value weighted aggregation on DNAm data in the post-integration dataset

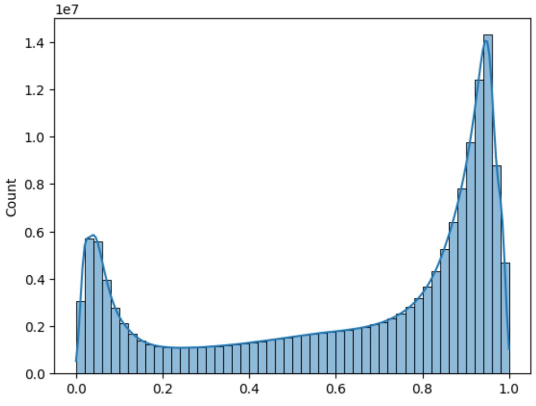

(a)

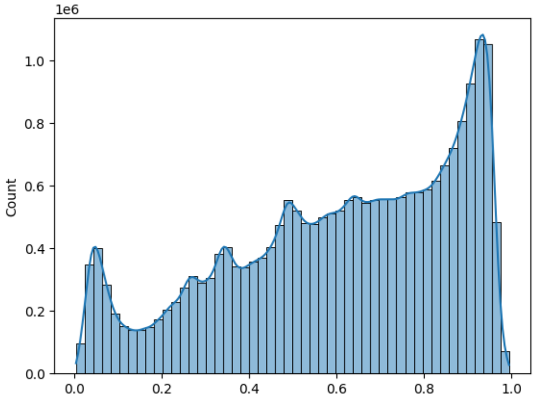

(b)

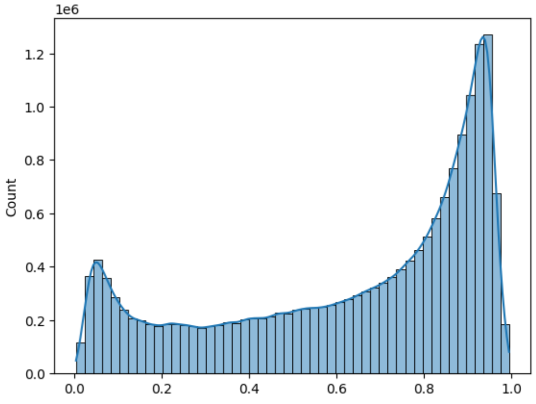

(c)

Figure S4: (a) shows the distribution of DNAm gene-body sites. (b) shows distribution of simple mean aggregation of gene-body methylation sites for each gene. (c) shows the distribution of p-value weighted aggregation of gene-body methylation sites for each gene.

Supplementary Figure S5. CNV-based SOPA compared to GSCA

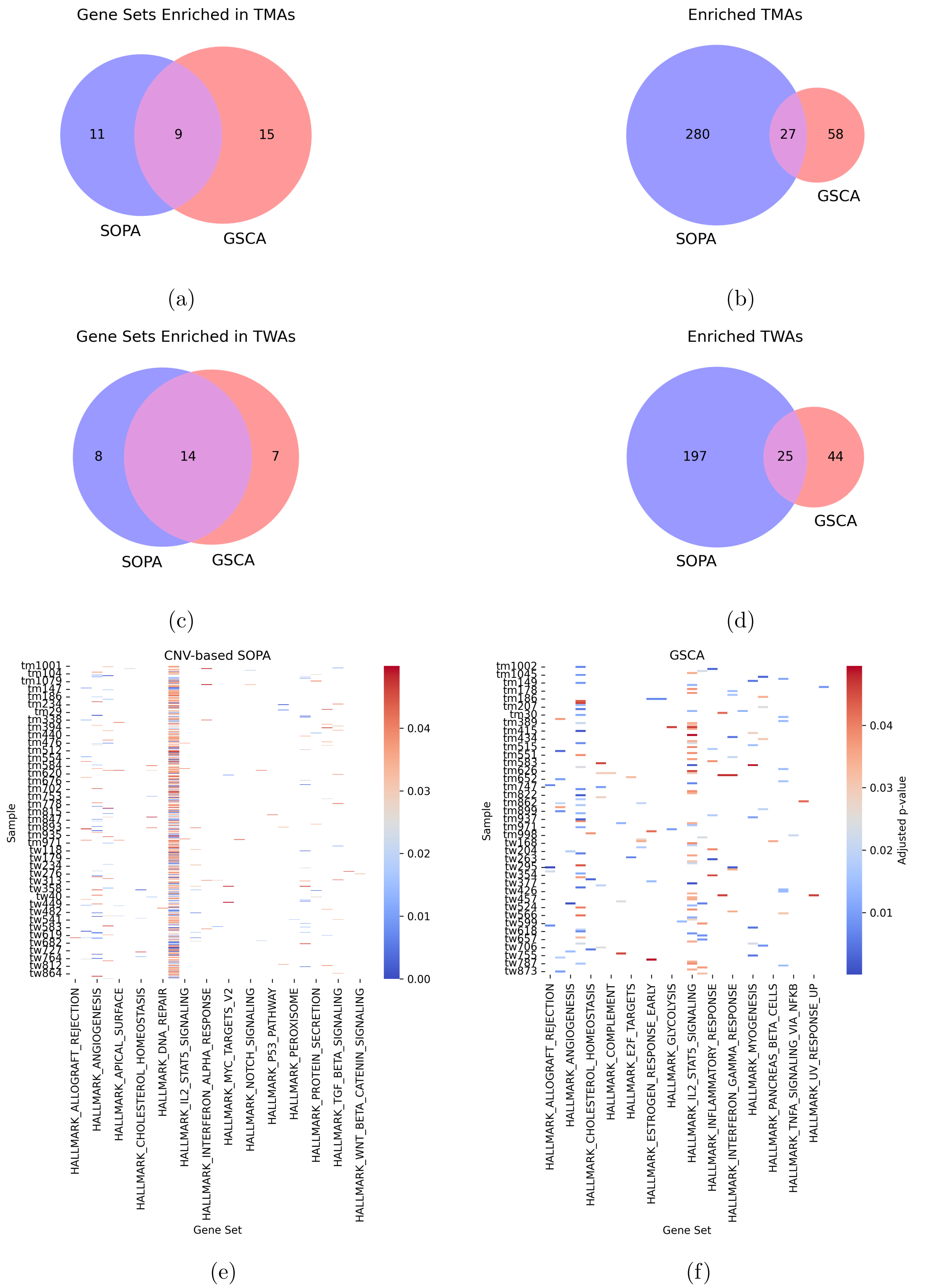

Figure S5: SOPA and GSCA similarity assessment. (Legend continues on next page)

Figure S5: (Figure legend continued from previous page) Significance threshold was considered to be FDR<0.05. (a) TMAs had 0.257 JI in terms of similar significantly enriched gene sets identified across CNV-based SOPA and GSCA, with 9 matching gene sets. (b) JI was around 0.0689 in enriched sample similarity. (c) As for TWAs, 14 gene sets were identified across the group by both SOPA and GSCA, with a JI of 0.483. Overall, JI between SOPA and GSCA across both TMAs and TWAs was 0.447. In the comparison when combining both groups, SOPA identified 27 gene sets and GSCA 28, with seventeen matching gene sets. (d) There were only 25 samples shared between both tools, resulting in JI = 0.084. SOPA identified significantly enriched genesets in 566 samples compared to 171 samples in GSCA. Overall, $W_{adjusted}(G_{x,s})$ is capable of identifying biological perturbations with acceptable capacity. Nonetheless, it is important to denote that results showed that sample-based similarity was low with a JI of 0.075. This is to be expected, as GSCA does not account for normalization against the TWA's CNV variation and does not rank genes. (e) GSCA identified significant enrichments in 171 samples after multiple testing adjustment. (f) CNV-based SOPA identified significant enrichments in 566 samples. These results indicate that the $W_{adjusted}(G_{x,s})$ ranking metric has improved inter-sample sensitivity in comparison to GSCA with multiple testing, providing evidence to its utility as a modality for single-sample pathway analysis.

Supplementary Figure S6. Group-based MSD and $MSD_{D_{x,s}}$

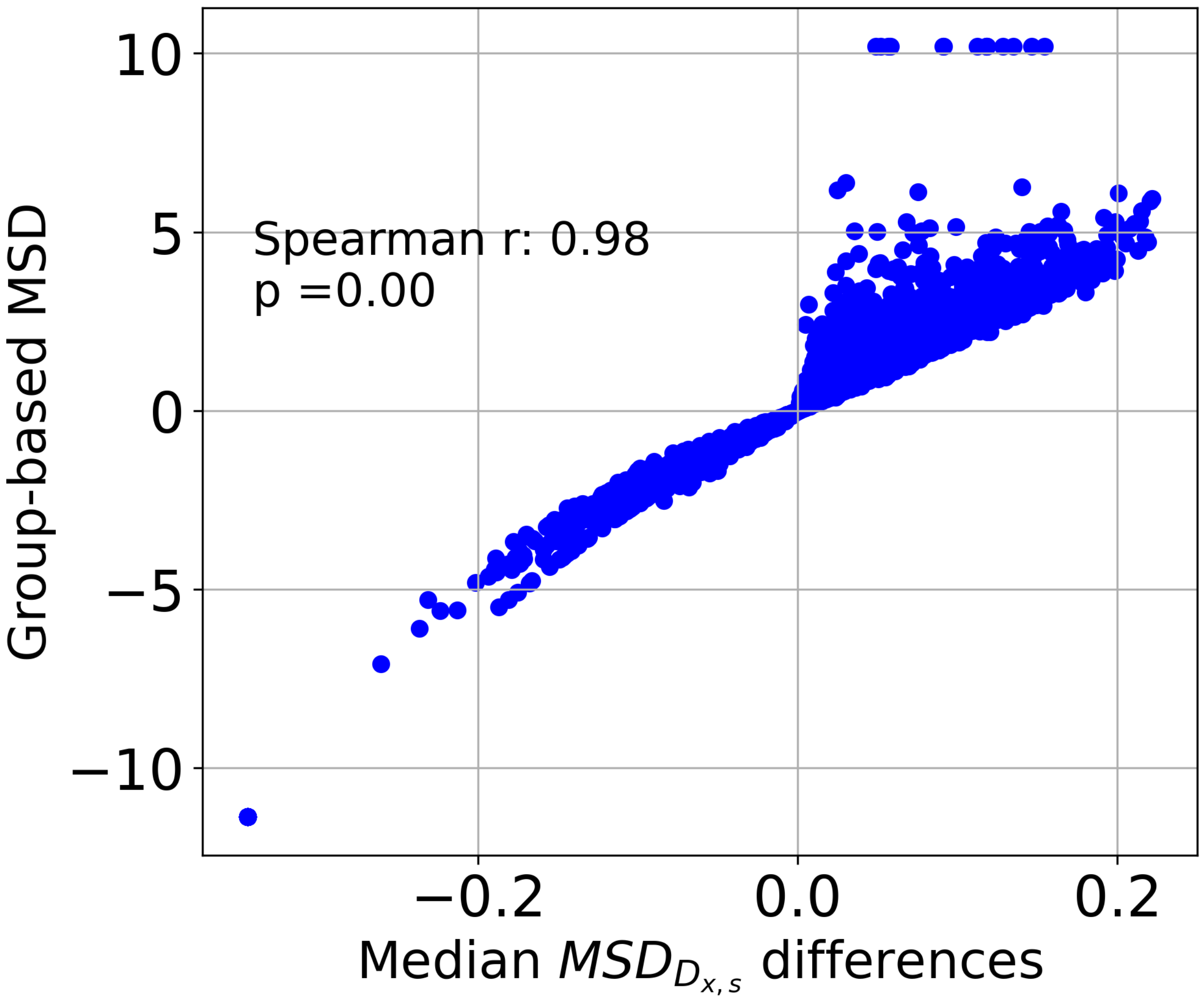

Figure S6: $MSD_{D_{x,s}}$ case-control aggregate median differences returned a Spearman's rank correlation coefficient of 0.98 ($p < 0.00005$) on RNAseq data, showing high concordance and validity of the metric when combined across all samples in comparison to classical group-based MSD.

Supplementary Figure S7. Group-based regression and $MSD_{D_{x,s}}$

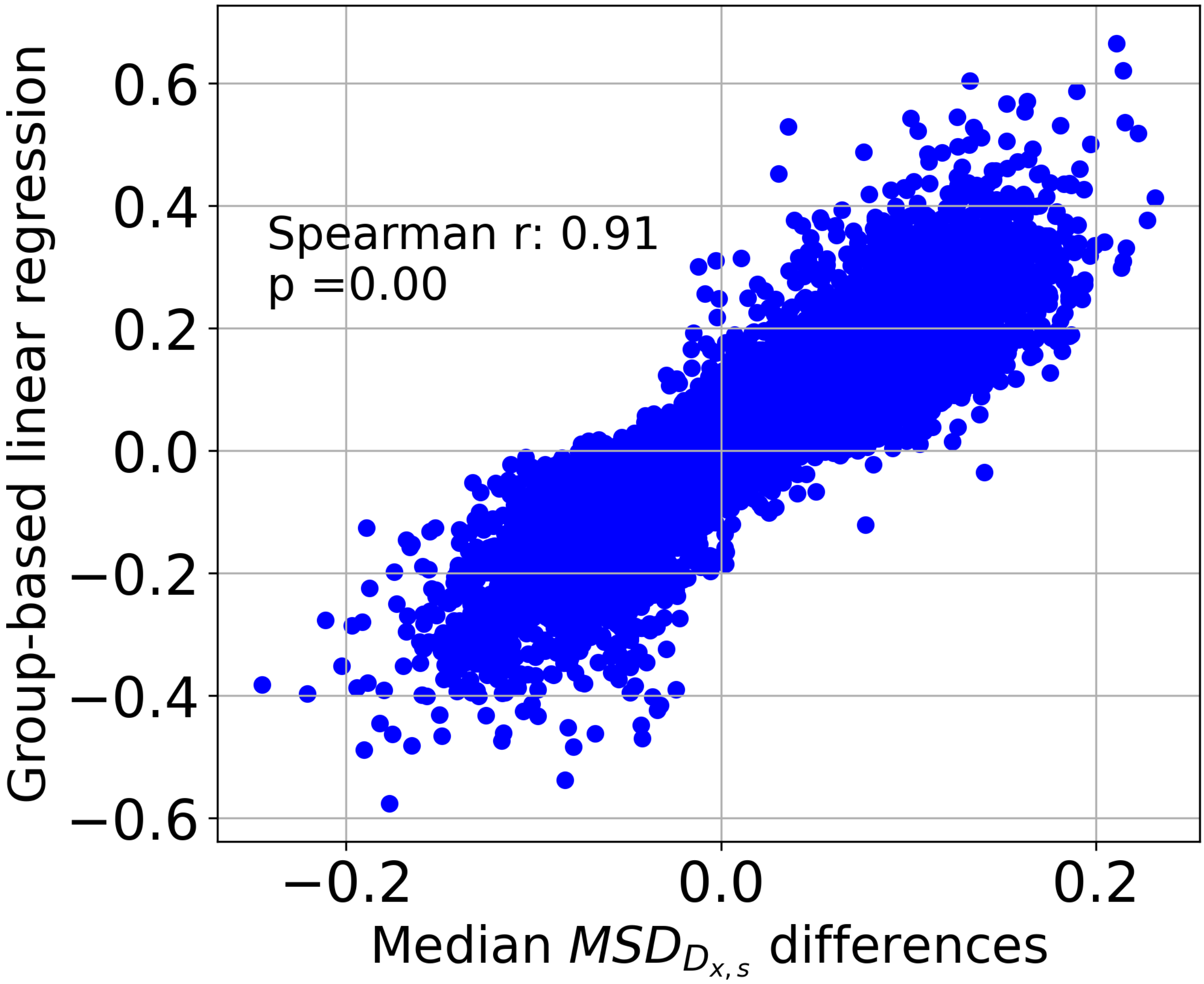

Figure S7: When assessing aggregate $MSD_{D_{x,s}}$ agreement with group-based linear regression, Spearman's coefficient returned a test statistic of 0.90 ($p < 0.00005$), indicating an even stronger relationship between the results of the two methods. As $MSD_{D_{x,s}}$ was superior, we utilized the metric in all of the subsequent analyses.

Supplementary Figure S8. Stability of SOPA ranking metrics

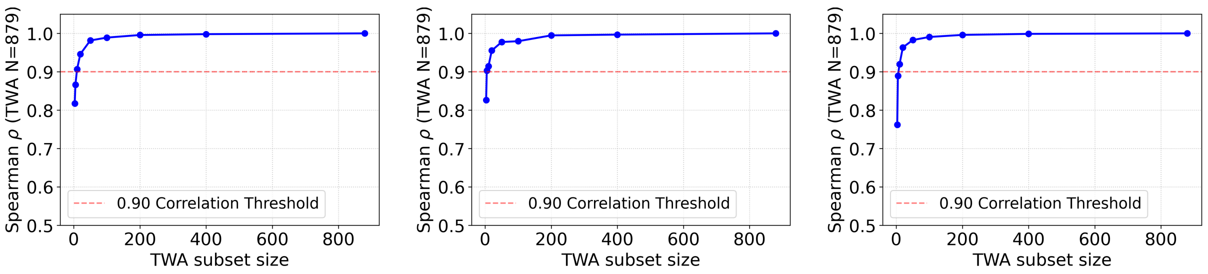

(a) (b) (c)

Figure S8: Control random subset downsampling for stability assessment of SOPA ranking metrics. Each dot represents the subset of random samples in the control group where TMAs were compared to. The subset number was increased per iteration of ρ recomputation in the following order: 3, 5, 10, 20, 50, 100, 200, 400, and 879 (TWAs). (a) $MSD_{D_{x,s}}$ for RNAseq data reaches the stability threshold at around 10 samples. (b) CNV-based $W_{adjusted}(G_{x,s})$ reaches stability at around 10 samples. (c) $MSD_{D_{x,s}}$ on DNAm data reaches stability at around 10 samples.

Supplementary Figure S9. SOPA, ssGSEA, and GSVA in RNAseq, CNV, and DNAm TCGA datasets

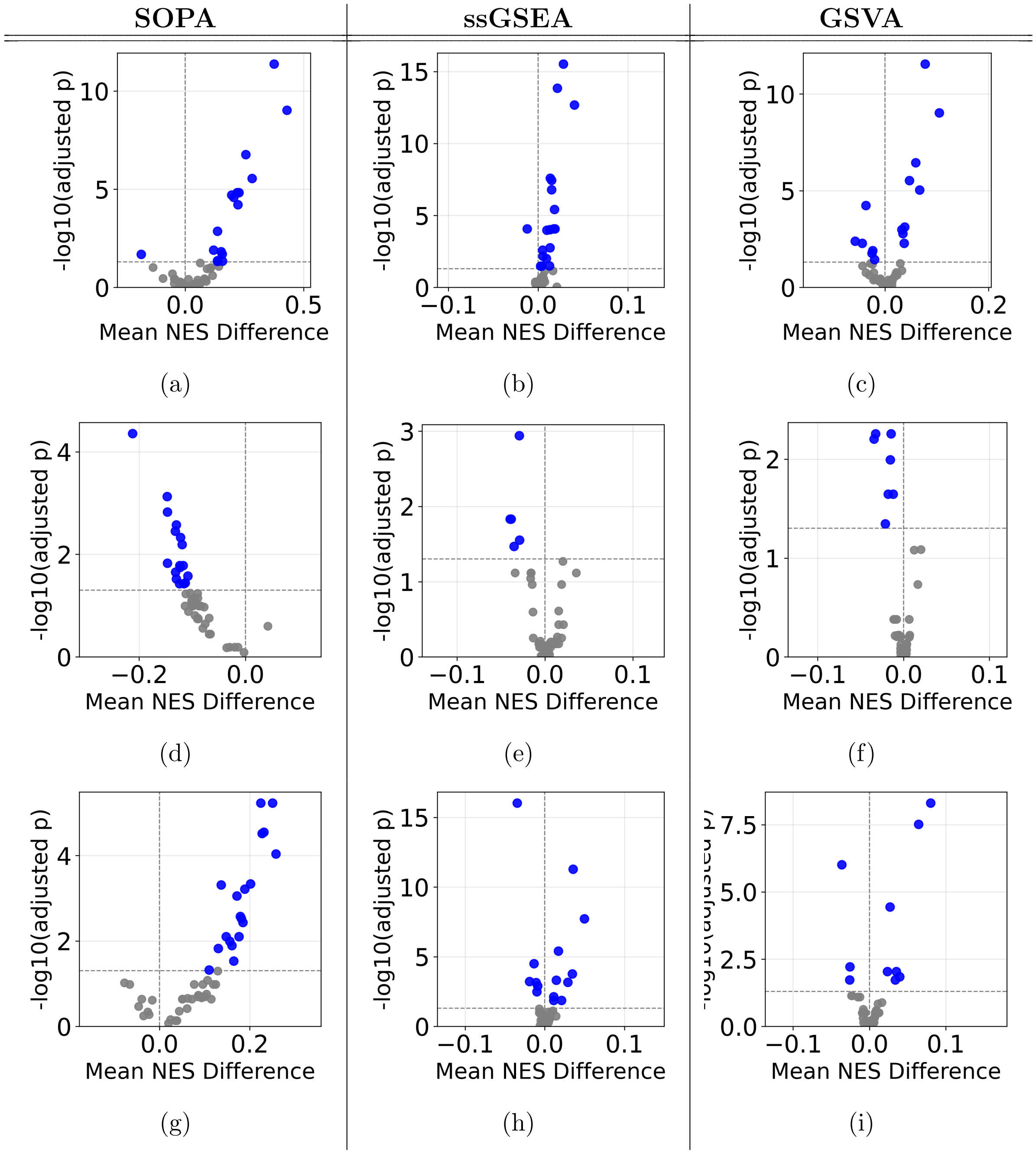

● BH-corrected $p < 0.05$
● Insignificant pathway differences observed by the tool

Figure S9: Comparison between SOPA, ssGSEA, and GSVA on datasets from TCGA for RNAseq, CNV, and DNAm datasets. (n=1,968) (Figure legend continues on next page)

Figure S9: (Figure legend continued from previous page) (a), (b), (c) Analyses on the RNAseq TCGA dataset using the three tools showed that SOPA was able to identify significant pathway deviations. (d), (e), (f) SOPA was able to determine a higher number of significantly differentiated pathways between TWAs and TMAs compared to ssGSEA and GSVA due to its custom ranking metric. (g), (h), (i) In the TCGA DNAm dataset, SOPA was similarly able to show group-based trends with more significantly differentiated pathways compared to other tools.

Supplementary Figure S10. TCGA supplementary comparison of SOPA, ssGSEA, and GSVA

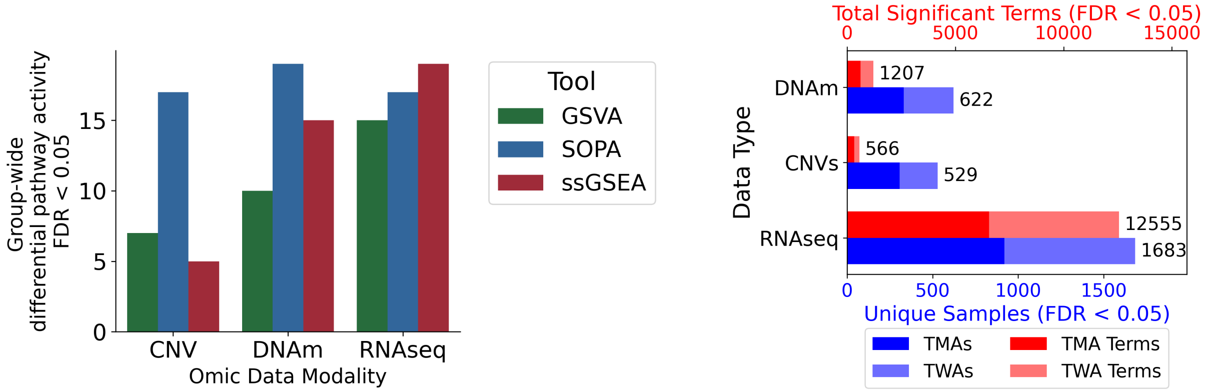

(a) (b)

Figure S10: Additional analyses comparing SOPA, ssGSEA, and GSVA across all –omics in the TCGA dataset. (n=1,968) (a) Significantly differentiated pathways between TWAs and TMAs across all –omics in the TCGA dataset. SOPA provides interpretability, with similar or improved performance compared to ssGSEA and GSVA. (b) Using SOPA to identify per-sample significantly differentiated pathways in reference to the control group across all –omics in the TCGA dataset. SOPA produces FDR values for each sample and pathway, using (by default) 1000 permutations, allowing for the identification of significantly enriched pathways in individual samples. On the other hand, both ssGSEA and GSVA produce enrichment scores that are not directly associated with p-values or FDR values for individual samples, making it difficult to identify significantly enriched pathways on a per-sample basis.

Supplementary Figure S11. Impact of control-normalization on NES in SOPA, compared to ssGSEA, and GSVA

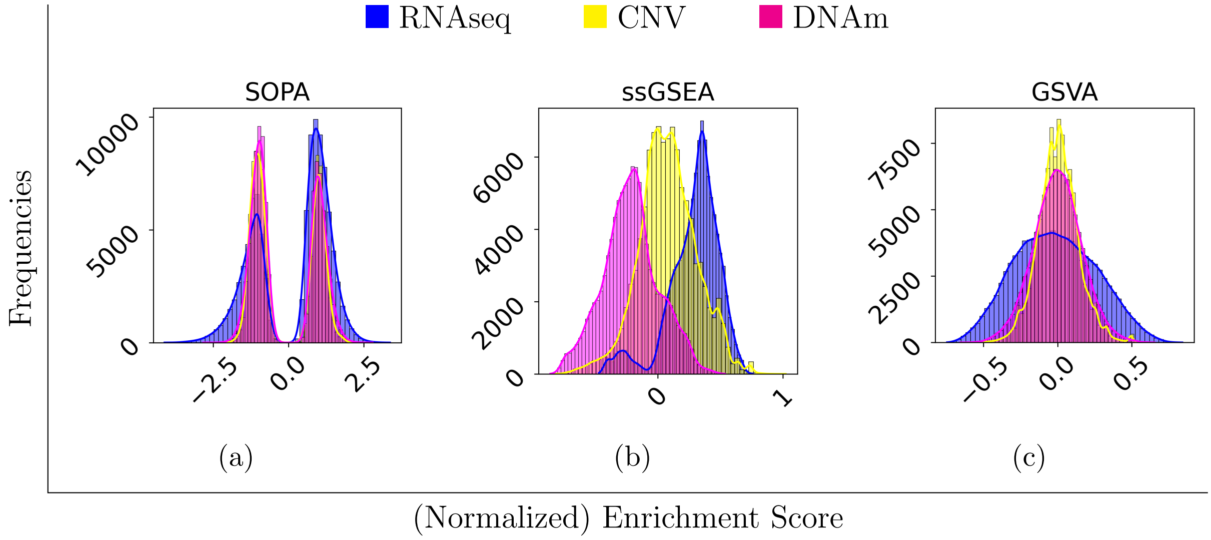

Figure S11: Impact of control-normalization on the calculated pathway activity scores obtained from SOPA, compared to ssGSEA and GSVA in the TCGA RNAseq, CNV, and DNAm datasets. (n=1,968) (a) SOPA has clear differentiation of positive and negative enrichment centered on 0 for all –omics. This indicates that samples are directly comparable, and the ranking metrics are effectively conveying differential pathway activity. (b) High variability is observed through ssGSEA between different –omics, indicating that the method is attempting to assess absolute pathway activity. (c) GSVA normalizes ES distribution across all –omics and centers values on 0. This indicates that the normalization is affecting all samples similarly without adjusting to the predefined controls. In both ssGSEA and GSVA, the algorithms compress NES and ES values towards 0, and resulting output is bounded in the range of -1 to 1, which reduces the variance of the scores and their interpretability.

Supplementary Figure S12. Results of MOGSA and padma analyses in the IM TCGA dataset

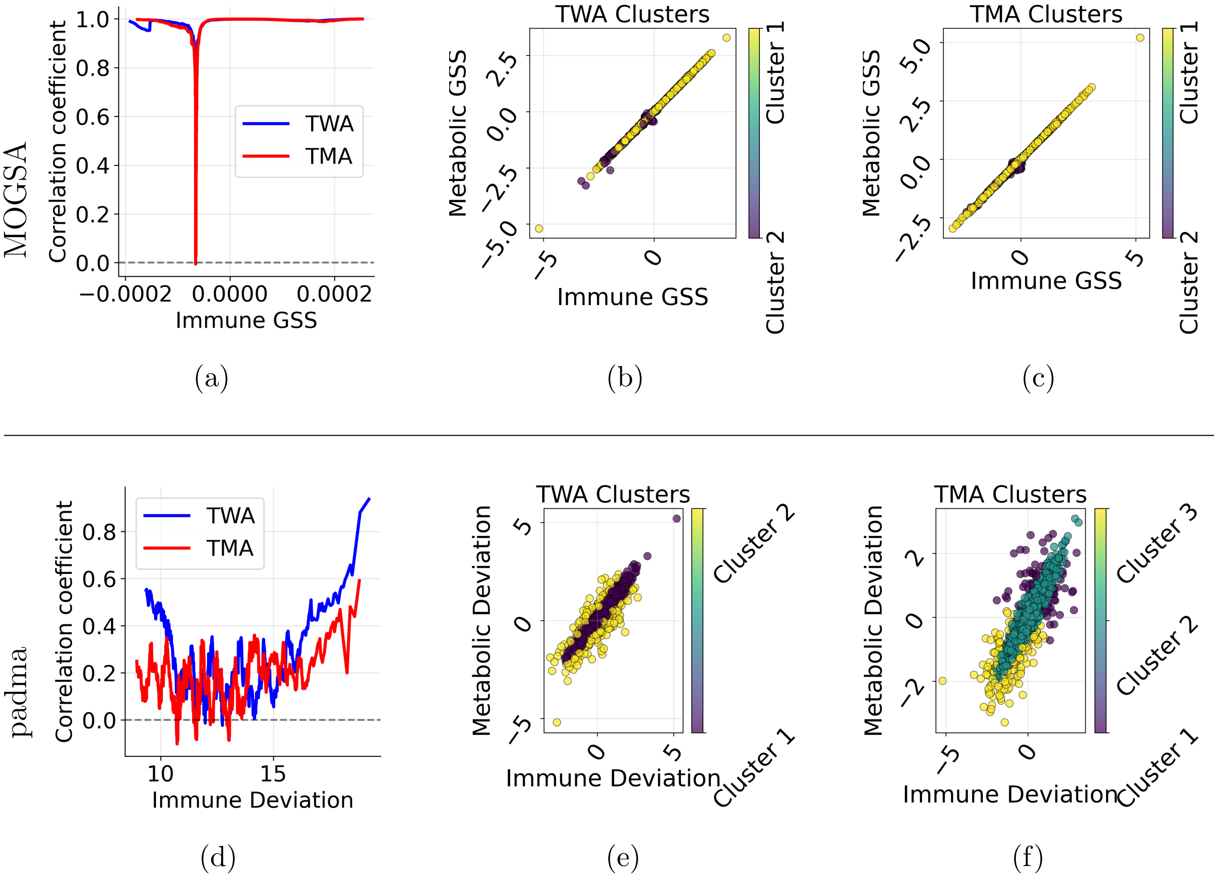

Figure S12: Supplementary analyses of MOGSA and padma results in the IM TCGA dataset. (n=1,968) (a) Sliding window correlation analysis shows that MOGSA was unable to identify unique divergence of immune and metabolic pathways between TWAs and TMAs, highlighting the importance of supervised analysis methodology in this context. (b), (c) Quantile normalized immune and metabolic GSS scores obtained by MOGSA did not provide clear separation to delineate intra-group heterogeneity. (d) Sliding window correlation analysis shows that padma was able to detect more divergence between TWAs and TMAs, due to its population-based approach. (e), (f) Quantile normalized deviation scores show more divergence compared to MOGSA, but do not provide stable subpopulation clusters.

Supplementary Figure S13. MOGSA determines RNA to be the most important component of the multiomics through PCA

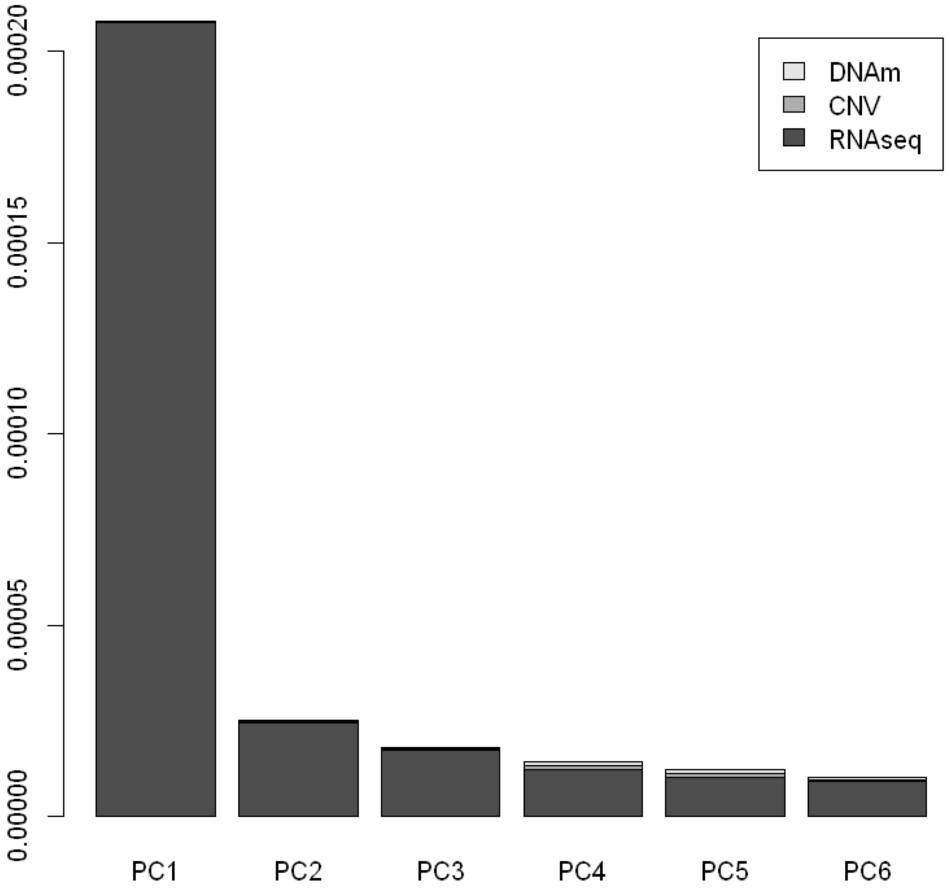

Figure S13: MOGSA identified that the highest variation in the data was in a single component, dominated by RNAseq values. X-axis contains principal components (PCs) learned by MOGSA. Y-axis represents the variance explained by each component in the data. To examine whether the inability to determine significant enrichment was due to the specific implementation of MOGSA, we analyzed the principal components of the integrated data. Multi-table analysis in MOGSA calculated that the main variance in the data was due to RNAseq, and that the components explain minimal shared insights between the components.

Supplementary Figure S14. Within-omic aggregation does not introduce bias in MPES scores

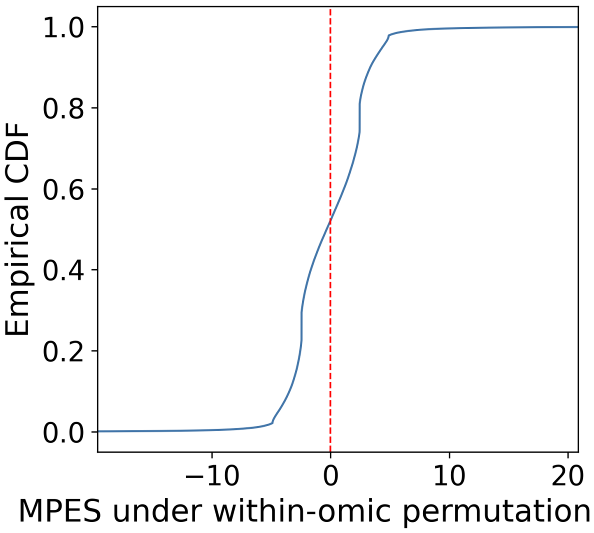

Figure S14: Aggregation-only null distribution of MPES. X-axis is clipped to the central 99.8% of the null distribution for improved visibility. Q005/Q995 are within the displayed range. The empirical cumulative distribution function (CDF) of MPES obtained from 10,000 random permutations for 50 pathways in 1,089 TMAs. For each TMA observed, terms were permuted within each –omic to perserve marginal distribution of WOCS values, removing cross-omic concordance. MPES was recalculated for each permutation, and the resulting distribution of MPES was plotted. The median for the null distribution was -0.3235, mean = -0.1133, IQR = (-2.449, 2.449), Q025/Q975 = (-4.879, 4.884), Q005/Q995 = (-8.920, 9.482). These results confirm that the WOCS aggregation does not introduce systematic bias of MPES scores. The IQR at (-2.449, 2.449) reflects expected mathematical properties of the MPES formula, where if two out of three random WOCS values are near equal, leading MPES to an expected value of $\pm\sqrt{6} \simeq \pm 2.449$.

Supplementary Tables

Table S1: Description of potentially relevant non-topology-based single sample pathway analysis tools for comparisons with SOPA, and selected tools for comparisons.

Tool	Array	Description	Disadvantages	Rationale for comparison
ssGSEA[4]	Transcriptomics. Can be potentially implemented in other array data	Original implementation uses absolute expression values to rank genes in single samples in a supervised manner	Does not allow for accurate differential pathway enrichment analysis. Does not control for "normal" samples. Does not test for false-positives.	- Included Notes: Original implementation does not return FDR values, and does not indicate significance of pathway enrichment. However, when using permutations, FDR values can be retrieved. Permutations can cause scores to be unstable.
GSVA[6]	Transcriptomics. Can be potentially implemented in other array data.	Performs a kernel density estimation of all samples to create a "normal" sample, which is used in comparison to every single sample in an unsupervised manner.	While allowing for differential pathway enrichment analysis, GSVA does not allow for supervised comparisons. Thus, NES values returned have little interpretability. Does not test for false-positives.	- Included Notes: The reason for inclusion is the potential for implementation in different single –omics data.

Continued on next page

Table S1 (continued)

Tool	Array	Description	Disadvantages	Rationale for comparison
PLAGE[7]	Transcriptomics	Uses singular value decomposition (SVD) to estimate normal pathway activity level based on the first ”meta-gene”, or gene with the highest impact, to estimate other genes’ importance	Does not account for a predefined control group. Does not perform multiple testing correction.	- Excluded Notes: Focuses on RNAseq data. Does not provide directly comparable results to the standard GSEA algorithm.
iPath[8]	Transcriptomics	Calculates gene rank based on absolute expression, and uses ranking to calculate individual enrichment score (iES) for pathways, which are compared to a ”normal sample”. Perturbed samples are assigned ”perturbed” status if a sample is 2 standard deviations far from the normal distribution of iES.	Unsupervised methodology that has only been validated on FPKM values. Uses a function to define a ”normal sample” for comparisons rather than a range of controls. Does not account for direction of pathway dysregulation.	- Excluded. Focuses on RNAseq data. Uses absolute within-sample gene normalization, rather normalizes pathways. Provides ”sample-wide” enrichment used for survival analysis. Focuses on patient stratification, rather than on calculating results based on stratified samples.

Continued on next page

Table S1 (continued)

Tool	Array	Description	Disadvantages	Rationale for comparison
Singscore[9]	Transcriptomics	Calculates individual gene scores by assessing the maximum and minimum gene expressions in a single sample.	Does not account for inter-sample heterogeneity.	- Excluded. Designed specifically for RNAseq.
Pathifier[10]	Transcriptomics. Can be potentially implemented in other array data.	Uses unsupervised principal curve analysis to project a "normal distribution" and assess single sample deviations from the estimated norm.	Does not allow for the use of predefined control groups. Does not implement multiple testing correction.	- Excluded. Focuses on assessing magnitude of pathway deregulation, rather than providing direction of dysregulation.
PASS[11]	Transcriptomics	Uses unsupervised network analysis to build sample-specific networks, which compares a single sample to a "healthy" sample. A fully connected network is additionally made for each pathway in each single sample.	Does not normalize single samples to a range of controls. Does not correct for multiple testing.	- Excluded. Focuses on RNAseq data. Focuses on classification of disease similarity.

Table S2: Information regarding used software and package versions. To conduct this research, python v3.11.9 was used in a jupyterlab notebook (v4.0.11) within an anaconda environment.

**Package**	**Utility**	**Version**
Dask	Package for managing large datasets	2024.9.1
Fancyimpute	Package to impute missing data	0.7.0
Gseapy	Allows conducting GSEA, ssGSEA, GSVA	1.1.3
imbalanced_learn	Required for conducting imputation	0.12.3
ipywidgets	Required for creating interactive plots in SIMPA	8.1.5
kaleido	Required for creating interactive plots in SIMPA	0.1.0.post1
Knnimpute	Allows KNN imputation	0.1.0
Lifelines	For survival and Kaplan-Meier analysis	0.29.0
Matplotlib	For creating images and plots	3.9.2
Numpy	For working with numerical data	1.23.5
Pandas	For managing datasets	2.2.2
pillow	Required for SIMPA image rendering	10.4.0
Plotly	Required for SIMPA image rendering	5.24.1
Pyarrow	Required for Dask	17.0.0
pydeseq2	To perform differential expression analysis	0.4.10
scikit-learn	To perform regression analysis	1.5.1
scikit-optimize	For training regression models	0.10.2
Scipy	For conducting statistical analyses.	1.14.0
Seaborn	To create images and plots	0.13.2
Statsmodels	Contains regression models and statistical analysis tests	0.14.2

Table S3: R packages used to utilize MOGSA and padma. R version used was 4.5.1, and the analyses were conducted in a local jupyter notebook.

**Package**	**Version**
BiocManager	1.30.25
dplyr	1.1.4
knitr	1.49
gplots	3.2.0
ggplots2	3.5.1
mogsa	1.40.0
readr	2.1.5
tidyverse	2.0.0
padma	1.22.0

Table S4: Computational specifications for SOPA ranking metrics and SIMPA.

**Component**	**Specification**
CPU	Ryzen 7600X
RAM	32GB DDR5

Table S5: Survival differences and demographics between TWAs and TMAs.

	TWA			TMA			
**Cancer Type**	**Count**	**Mean**	**STD**	**Count**	**Mean**	**STD**	**p-value**
Lung	517	939.93	831.31	453	729.85	618.01	<0.0001
Breast	413	1958.51	1336.11	583	1459.12	1313.20	<0.0001
Colon	223	998.80	950.02	175	556.96	499.74	<0.0001
Thyroid	153	1042.67	694.91	230	957.89	636.33	0.3687
Pancreas	42	632.84	438.08	248	508.78	383.73	0.1042
Kidney	95	1042.41	597.88	54	597.13	512.52	<0.0001
Overall Summary Statistics							
**Metric**	**Count**	**Mean**	**STD**	**Count**	**Mean**	**STD**	
Age (Years)	1443	61.86	13.43	1743	58.59	14.43	–
Survival (Days)	1443	1046.3	957.2	1743	766.6	788.9	<0.0001

Table S6: Effect size differences are reported as median $|\delta|$ of all pathway differences observed by the tools.

**Tool**	**–omic**	$median|\delta|$	**CI max**	**CI min**	$|\delta| \geq 0.147$
**SOPA**	RNAseq	0.038	0.244	-0.122	3
**SOPA**	CNV	0.053	0.089	-0.175	0
**SOPA**	DNAm	0.051	0.187	-0.105	0
**ssGSEA**	RNAseq	0.038	0.277	-0.163	6
**ssGSEA**	CNV	0.024	0.122	-0.159	0
**ssGSEA**	DNAm	0.045	0.242	-0.280	3
**GSVA**	RNAseq	0.041	0.244	-0.169	2
**GSVA**	CNV	0.026	0.118	-0.150	0
**GSVA**	DNAm	0.037	0.220	-0.192	2

Median $|\delta|$ was calculated by pooling all results between TWAs and TMAs across all pathways. CI max and min were calculated through bootstrapping with 1000 iterations, and represent the maximum and minimum values of the 95% confidence interval for the median $|\delta|$. If a tool's output had a Cliff's delta $|\delta|$ value higher than 0.147 it was considered to have a "non-negligible" effect size, and the number of pathways with $|\delta| \geq 0.147$ was calculated. No tool had an output with $|\delta|$ value higher than 0.33 (no effect size differences beyond "small" or "negligible").

Table S7: MOGSA confirms low correlations in the integrated dataset. Table shows RV coefficients for pairwise closeness obtained through MOGSA. The difference in data types found in the integrated omics is large, and represents different aspects of molecular structures, all of which were obtained through different platforms. This poses a difficulty to MOGSA to identify patterns consistent across the multiomics. RV coefficients calculated by MOGSA validate our low Pearson's correlation coefficients, and are strong evidence to the global low correlations between the multiomics analyzed.

	**RNAseq**	**CNV**	**DNAm**
**RNAseq**	1.00000	0.08590450	0.48015413
**CNV**	0.0859045	1.00000	0.09165
**DNAm**	0.4801541	0.08994229	1.00000

Table S8: Gene coverage loss and functional categorization of lost genes resulting from gene-centric multiomics integration. The table shows the number of unique genes lost through integration on aligned samples (n=1,968). Top section shows the total number of genes covered by each –omic array, before and after integration. Middle section shows the number of lost genes per –omic across each functional category. Bottom section shows the percentage of lost genes per total genes in the specific functional category per –omic. Methylation arrays retain most protein-coding genes, due to the platform design preferentially targeting protein-coding gene bodies.

**Omic**	**Gene coverage**	**Genes retained after integration**	**% retained**
RNAseq	60660	9142	15.07%
CNV	55820	9142	16.38%
DNAm (gene-level, gene-body)	11369	9142	80.41%
**Functional categories lost through integration on aligned samples (n=1,968)**			
**Functional Category**	**RNAseq**	**CNV**	**DNAm**
lncRNA	16827	15974	8
pseudogene	15166	13332	6
misc_RNA	2211	2065	0
snRNA	1901	1760	0
miRNA	1817	1628	7
TEC	1057	1001	0
snoRNA	933	880	0
All other non-coding/specialized	542	402	1755
protein_coding	11064	9636	451
**% lost of functional category per –omic**			
Non-coding % lost	99.40%	99.35%	87.92%
protein_coding % lost	55.43%	51.99%	4.82%

Supplementary References

[1] Edge SB, Compton CC. The American Joint Committee on Cancer: the 7th edition of the AJCC cancer staging manual and the future of TNM. Ann Surg Oncol. 2010 Jun;17(6):1471-4. doi: 10.1245/s10434-010-0985-4

[2] Canzler, S., and Hackermüller, J. (2020). multiGSEA: a GSEA-based pathway enrichment analysis for multi-omics data. *BMC Bioinformatics* 21, 561. 10.1186/s12859-020-03910-x.

[3] Subramanian, A., Tamayo, P., Mootha, V.K., Mukherjee, S., Ebert, B.L., Gillette, M.A., Paulovich, A., Pomeroy, S.L., Golub, T.R., Lander, E.S., and Mesirov, J.P. (2005). Gene set enrichment analysis: A knowledge-based approach for interpreting genome-wide expression profiles. *Proceedings of the National Academy of Sciences* 102, 15545-15550. 10.1073/pnas.0506580102.

[4] Barbie, David A et al. "Systematic RNA interference reveals that oncogenic KRAS-driven cancers require TBK1." *Nature* vol. 462,7269 (2009): 108-12. doi:10.1038/nature08460

[5] Fang, Z., Liu, X., and Peltz, G. (2023). GSEApy: a comprehensive package for performing gene set enrichment analysis in Python. *Bioinformatics* 39, btac757. 10.1093/bioinformatics/btac757.

[6] Hänzelmann, S., Castelo, R., and Guinney, J. (2013). GSVA: gene set variation analysis for microarray and RNA-Seq data. *BMC Bioinformatics* 14, 7. 10.1186/1471-2105-14-7.

[7] Tomfohr, J., Lu, J., and Kepler, T.B. (2005). Pathway level analysis of gene expression using singular value decomposition. *BMC Bioinformatics* 6, 225.

[8] Su, K., Yu, Q., Shen, R., Sun, S.-Y., Moreno, C.S., Li, X., and Qin, Z.S. (2021). Pan-cancer analysis of pathway-based gene expression pattern at the individual level reveals biomarkers of clinical prognosis. *Cell Reports Methods* 1, 100050. 10.1016/j.crmeth.2021.100050.

[9] Foroutan, M., Bhuva, D.D., Lyu, R., Horan, K., Cursons, J., and Davis, M.J. (2018). Single sample scoring of molecular phenotypes. *BMC Bioinformatics* 19, 404.

[10] Drier, Y., Sheffer, M., and Domany, E. (2013). Pathway-based personalized analysis of cancer. *Proceedings of the National Academy of Sciences* 110, 6388-6393.

[11] Li, X., Li, M., Zheng, R., Chen, X., Xiang, J., Wu, F.-X., and Wang, J. (2020). Evaluation of Pathway Activation for a Single Sample Toward Inflammatory Bowel Disease Classification. *Frontiers in Genetics* 10, 1374.
